# Isolation of novel citrus and plum fruit promoters and their functional characterization for fruit biotechnology

**DOI:** 10.1186/s12896-020-00635-w

**Published:** 2020-08-20

**Authors:** Kasturi Dasgupta, Sara Hotton, William Belknap, Yasra Syed, Christopher Dardick, Roger Thilmony, James G. Thomson

**Affiliations:** 1 Citrus Research Board, Visalia, CA USA; 2 Crop Improvement and Genetics, Western Regional Research Center, USDA-ARS, Albany, CA USA; 3 Present address: Impossible Foods, Redwood City, CA 94063 USA; 4 Genetic Improvement of Fruit Crops using advanced Genomics and Breeding Technologies, Kearneysville, WV USA

**Keywords:** Promoter, Fruit-specific expression, Citrus (*Citrus sinensis*), Tomato (*Solanum lycopersicum*), Plum (*Prunus americana*), Transgenic

## Abstract

**Background:**

Promoters that confer expression in fruit tissues are important tools for genetic engineering of fruit quality traits, yet few fruit-specific promoters have been identified, particularly for citrus fruit development.

**Results:**

In this study, we report five citrus fruit-specific/preferential promoters for genetic engineering. Additionally, we have characterized a novel fruit-preferential promoter from plum. Genes specifically expressed in fruit tissues were selected and their isolated promoter regions were fused with the *GUSPlus* reporter gene for evaluation in transgenic plants. Stable transformation in Micro-Tom tomato demonstrated that the candidate promoter regions exhibit differing levels of expression and with varying degrees of fruit specificity.

**Conclusions:**

Among the five candidate citrus promoters characterized in this study, the *CitSEP* promoter showed a fruit-specific expression pattern, while the *CitWAX* and *CitJuSac* promoters exhibited high fruit-preferential expression with strong activity in the fruit, weak activity in floral tissues and low or undetectable activity in other tissues. The *CitVO1, CitUNK* and *PamMybA* promoters, while exhibiting strong fruit-preferential expression, also showed consistent weak but detectable activity in leaves and other vegetative tissues. Use of these fruit specific/preferential promoters for genetic engineering can help with precise expression of beneficial genes and help with accurate prediction of the activity of new genes in host fruit plants.

## Background

Fruit are important sources of nutrients, minerals, vitamins, and dietary fiber, and as such, significant efforts have been made to breed for fruit with higher yield and better quality. Traditional methods of fruit breeding have been hampered by a number of challenges, including large plant size, long juvenile phase, and limited genetic gains [1, 2]. Use of genetic enhancement of fruit crops, has focused mainly on enhancing disease resistance (viruses, fungi, and bacteria), increasing tolerance to abiotic stresses (drought, frost, and salt), and modifying plant growth habit and fruit quality [3]. As such, there are few cases of field evaluation and commercial application of these improvements in transgenic trees [4–7]. The use of tissue-specific promoters is critical for producing transgenic crops with improved economically important tissues, such as the fruits in citrus. Citrus is one of the most important fruit crops worldwide and global demand is continuously on the rise [8, 9]. The fruit are considered healthy foods due to being low in fat and protein and rich in dietary fiber, vitamin C, B vitamins, (thiamin, pyridoxine, niacin, riboflavin, pantothenic acid, and folate), vitamin A (β-cryptoxanthin) carotenoids, flavonoids, and limonoids [9–11]. These biological active compounds are important to human health for their antioxidant quality and protection from various chronic diseases [8]. Recently, transgenic approaches to engineer improved citrus plants have gained importance mainly due to the rise of citrus greening disease huanglongbing (HLB) and the difficulty in controlling it [12–14].

Transgene expression can be beneficially controlled by using promoters that are suitable to the plants’ genetic background, type of transgene expressed and/or the desired trait [15, 16]. Moreover, precise spatial (specific expression at the organ, tissue and cell levels) and temporal (developmental stage) expression of the transgene can minimize potential adverse effects or over-taxing the plant by producing unneeded products throughout the plant [17]. The use of computational algorithms to identify promoters in plant genomes is expected to further enhance the number of newly identified promoters from different species [10, 15]. Promoters that are capable of driving fruit-specific or fruit-preferential expression would be valuable tools for engineering improved fruit traits, such as better growth, ripening, nutritional quality, and post-harvest shelf life. To date, a number of fruit-specific promoters have been isolated and characterized from various plant species. For instance, the tomato *E4* and *E8* promoters have been found to be fruit-specific and are coordinately regulated by ethylene during fruit ripening [18, 19]. Additional tomato fruit-specific promoters include the promoter of the polygalacturonase gene (*PG*), which plays a role in cell wall degradation during fruit ripening [20–23]; the promoter of the *T-proline-rich protein F1* (*TPRP-F1*) gene, which is specifically expressed in the ovary and young fruit [24]; and the promoter of *ACC synthase* [25, 26]. A few fruit-specific promoters have also been isolated from non-tomato plant species, such as the ripening-upregulated gene *ACC-oxidase* in apple and peach, and the *expansin* promoter from sour cherry [25–27]. However, in many fruit species such as citrus and plum, the availability of fruit-specific promoters suitable for use in genetic engineering is still limited.

Plums are important stone fruits worldwide with production reaching nearly 20 million tons a year according to FAO data (http://www.fao.org/faostat/en/). However, plant diseases such as plum pox continues to be a major threat and is considered as one of the most serious viral disease of stone fruit trees [28]. Conventional breeding of plum requires long reproductive cycle with long juvenile periods and complex reproductive biology leading to the use of biotech approaches for development of resistance. With the availability of a high throughput transformation system, plum has become a model functional genomic system due to the recalcitrance of most fruit crop species [29].

Recently, advances in genomics and genetic engineering have been used to improve fruit crops by affording new sources of desirable characteristics and shortened breeding cycles [30–33]. Genetic engineering has emerged as a significant tool for the improvement of perennial tree species including fruit trees such as citrus in which development of new cultivars is often constrained by their long generation time, high heterozygosity, nucellar embryony and other reproductive barriers [4, 6]. Genomic manipulation also allows the addition of novel traits while maintaining specific cultivar traits desired but often lost or changed during traditional breeding.

Identification of novel promoters that provide robust and reliable fruit expression for use in engineering citrus for improved agronomic traits and nutritional quality will provide useful tools for fruit trait modification. In the present study, novel citrus and plum promoters were isolated and analyzed for their ability to be used as independent functional elements conferring transgene expression in fruit tissues. Because the effort required for the stable transformation in citrus and the long juvenile period delaying fruit production and analysis, the activities of the selected candidate promoters were examined in transgenic tomato, an important model system for fruit development research.

## Results

### Identification of candidate fruit-specific/fruit-preferential promoters

**Fig. 1:**
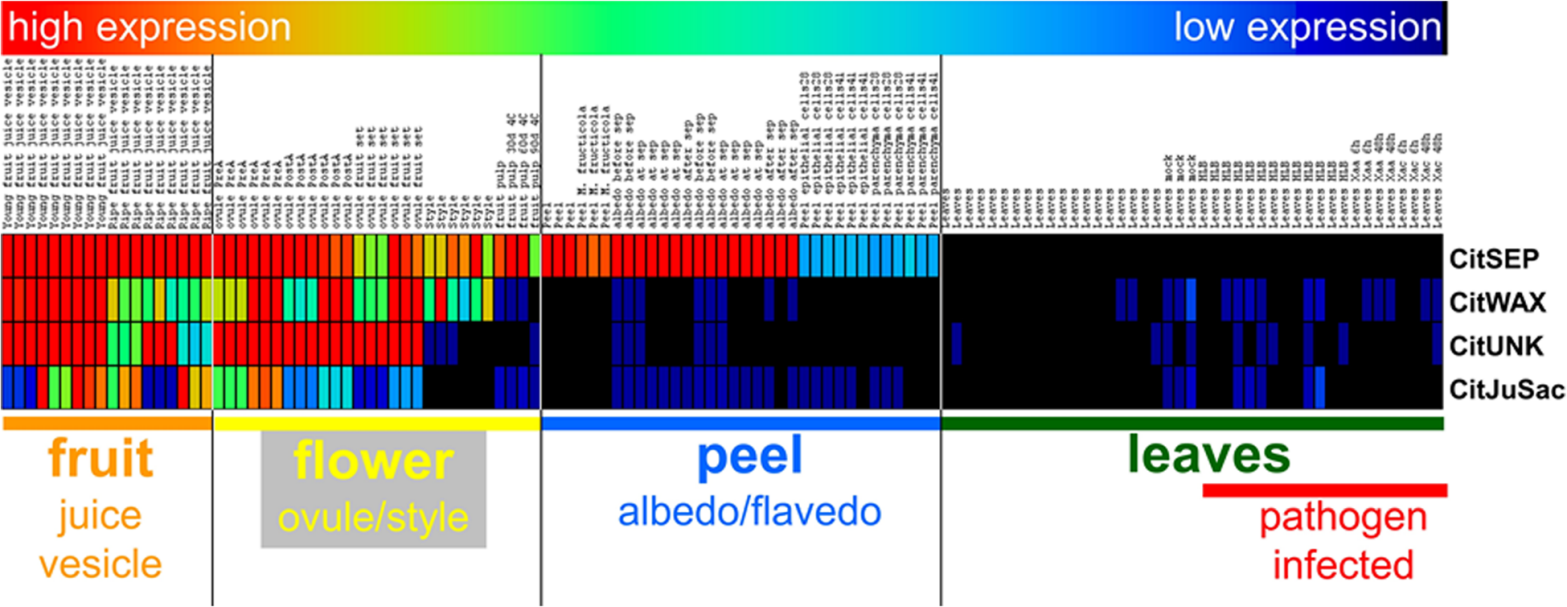
Candidate citrus gene expression patterns in different tissues. A heatmap displaying publicly available citrus gene expression data for four selected candidate genes is shown. The normalized expression levels are colored according to the scale bar shown on the top of the image. Each column represents a single biological replicate of the indicated sample, each row represents the candidate gene. The four candidate citrus genes were selected based on their high-level of expression in fruits and flowers compared to low or undetectable expression in leaves

Public citrus gene expression data available from the Gene Expression Omnibus (GEO) data repository (www.ncbi.nlm.nih.gov/geo) and PlexDB (https://www.bcb.iastate.edu/plant-expression-database) databases [34, 35] was analyzed to identify potential candidates. Candidate citrus genes that expressed in various tissues of the orange fruit (juice vesicles, albedo and flavedo), but exhibit little or no expression in vegetative tissues (i.e. leaves, roots etc.) were identified. The selected genes exhibited high levels of transcript in fruit tissues and sometimes flower tissues but had little or no detectable expression in leaves or other vegetative tissues (Fig. 1; Table 1).

**Table 1:** Citrus candidate fruit-specific genes

Candidate	Gene ID	Affymetrix Probe Set ID	GenBank Accession	Gene Homology
*CitSEP*	Cs7g10980	Cit.144.1.S1_s_at Cit.29312.1.S1_s_at	CB293157	Sepallata3 MADS-box protein 4
*CitWAX*	Cs1g02750	Cit.11241.1.S1_s_at	CX049273	Aldehyde decarboxylase, WAX2, CER1 fatty acid hydroxylase
*CitUNK*	Cs5g31450	Cit.29634.1.S1_at	CK935639	Unknown
*CitJuSac*	Cs6g16160	Cit.12380.1.S1_at	CF509979	*Cl111* juice sac

The *CitSEP* candidate (Cs7g10980) encodes a Sepallata 3 MADS-box like protein (Table 1). Sepallata 3 is a member of the SEP subfamily of MADS-box genes, whose members have nearly redundant functions in the specification of floral meristem identity in sepals, petals, stamens, and carpels [36]. The first intron of the *Arabidopsis SEP3* gene was shown to be important for floral expression specificity [36] thus, we isolated the 3406 bp sequence upstream of the *CitSEP* start codon including the first intron of the gene for testing. The *CitWAX* candidate encodes a protein with aldehyde decarboxylase/WAX2/CER1 fatty acid hydroxylase homology, suggesting a potential role in fatty acid metabolism in citrus fruit (Cs1g02750; Table 1)*. CitUNK* (Cs5g31450; Table 1) was the third selected candidate, this gene encodes a protein of unknown function. The orientation of this gene in the genome was determined based on presence of a poly-A tail on a corresponding cDNA, despite this orientation being in conflict with the annotated *C. sinensis* genome sequence. The fourth selected candidate, *CitJuSac* (Cs6g16160; Table 1) is a sweet orange homolog of the juice sac-specific *Cl111* gene, whose promoter was previously isolated from acid lemon *Citrus limon* L. and acidless lime *C. limettioides* Tan [37].. The *CitJuSac* gene is predicted to encode a dihydrofolate reductase/serine hydrolase protein potentially involved in the tetrahydrofolate biosynthesis metabolic pathway.

The *CitVO1* gene was selected based on its transcript being abundant in fruit/flower citrus Expressed Sequence Tag (EST) libraries (the abundance of its cDNA sequences in fruit and floral EST libraries was much higher than in libraries from other tissues). This promoter belongs to the *SCAmpPs* gene family in citrus [38]. The *SCAmpPs* genes are present in clusters within the citrus genome, in association with genes encoding receptor leucine-rich repeat proteins. The SCAmpPs amino acid composition, protein structure, expression patterns, evolutionary profile and chromosomal distribution are consistent with designation as ribosomally synthesized defense-related peptides. The *CitVO1* gene was not represented in the microarray expression data due to its small size, thus results for this gene are not shown in Fig. 1. The coding sequence for *CitVO1* is small and it contains a small intron and since introns have been shown to contribute to gene expression, the native intron along with promoter sequence was cloned and tested. An alternate version of the *CitVO1* promoter, lacking the native intron, was also cloned and designated *CitVO2*. The seventh candidate promoter we tested was from the feral plum *PamMybA*. *PamMybA* has been shown to activate anthocyanin production in transgenic plants [39] and was chosen due to its presumed expression during fruit ripening. The promoter sequences of these seven candidate genes are hereinafter referred to by their gene name addended with “p” to denote promoter.

### Isolation of the promoter sequences and analysis of their *cis*-elements

**Fig. 2:**
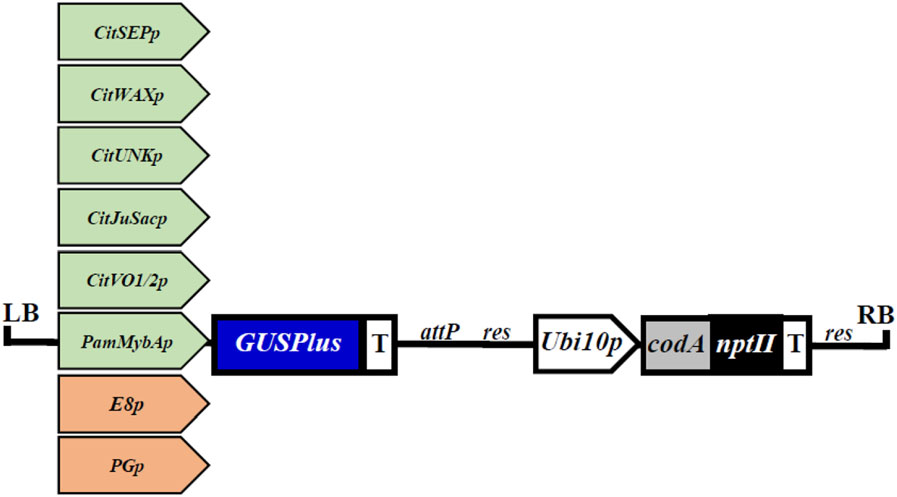
Schematic representation of the promoter-*GUSPlus* construct T-DNA. The T-DNA of the pCTAGII-GUSPlus binary vector is shown. It harbors a *codA-nptII* fusion gene selection marker under the control of the *Arabidopsis Ubiquitin10* promoter (*Ubi10p*) and nopaline synthase terminator (T). Candidate fruit-specific promoters from citrus (*CitSEPp*, *CitWAXp*, *CitUNKp*, *CitJuSacp, CitVO1*) and plum (*PamMybAp*), as well as control fruit-specific promoters from tomato (*E8p* and *PGp*) are shown. The T-DNA also contains *attP* and *res* recombination recognition sites for the Bxb1 and CinH recombinase enzymes respectively. LB and RB designate the *Agrobacterium* left and right borders

The promoters of the selected candidate genes were identified using the available citrus genome sequences (http://citrus.pw.usda.gov/, http://www.phytozome.net/citrus.php, www.citrusgenomedb.org/) and approximately 1–3 kilobase pair region upstream of the translation initiation codon (ATG). The candidate promoters were PCR amplified from sweet orange or plum genomic DNA (see Materials and Methods; Table 2) and cloned into the pCTAGII-GUSPlus vector (GenBank MG818373) fused to the reporter gene (Fig. 2). In addition to the citrus and plum candidate promoters, two control fruit-specific promoters from tomato, *E8* (*E8p*), and *PG* (*PGp*) were also fused to *GUSPlus* in the pCTAGII binary vector [18–21]. The candidate promoter sequences are available in Genbank under the following accession numbers. *CitSEPp* (MK012379), *CitWAXp* (MK012380), *CitUNKp* (MK012381), *CitJuSacp* (MK012382), *CitVO1p* (MK012383), *CitVO2p* (MK012384), *PamMybAp* (MK012380).

**Table 2:** Primers used for candidate promoter isolation

Promoter	Primer	Sequence 5′ to 3′
*CitSEPp*	CitSEP_FOR1_KpnISbfI 61	ttttGGTACCCCTGCAGGGCCATGGGAGAAGGTGCACATACTTTAG
	CitSEP_REV1_XmaIClaI55	ttttCCCGGGATCGATTTTCTTCTCCTTTCTTTCTTCTTCTATCAC
	CitSEP_INT FOR2 ClaI54	ttttATCGATCTCCAATAGAGGAAAGCTGTACG
	CitSEP_REV10_NotIPmeI55	ttttGCGGCCGCGTTTAAACGTTGCACTTCTGGTACCTCTC
*CitWAXp*	CitWAX_REV1_NcoI61	tttCCATGGTGCACTTTGAGGTAATGCAACATGCAATTGCTAG
	CitWAX_INT FOR2_EcoRI 59	tttGAATTCGAGAGGAAGAGAACAACAAATTAATAAAGGCGG
*CitUNKp*	CsUNK_FOR EcorI58	aaaaGAATTCCCTCAATCTGCACCACTAAGACGAAT
	CsUNK_REV NcoI59	aaaaCCATGGTTGTCTGTGGCATTCACTGGAGAG
*CitJuSacp*	CitSin JuSac_FOR2 EcoRI 52	tttGAATTCGAGAGGAAGAGAACAACAAATTAATAAAGGCGG
	CitSin JuSac_REV1 NcoI 53	tttCCATGGTTTTTTCTATTTCATTCTTTCAGATTTTAAGC
*CitVO1/2p*	CsCandidate#6_FOR EcoRI55	aaaaAACAAACTCCGCATAGTGGTTA
	CsCandidate#6_REV2 NcoI56	aaaaAAGATTTCTGCTGGCTTCGAC
	CsCandidate#6_REV1 NcoI56	aaaaCCGACCAATCGGTATAACCTGAA
*PamMybAp*	PfeMybA SbfI F61	agtcCCTGCAGGGATTTTCCACCTAATTGCACATCGATCCAAACG
	PfeMybA NcoI R59	agtcCCATGGTTTTCTTTTGGGCAGCGTTGTATGCTTGCAGC

The promoter sequences were analyzed and the presence of known *cis* elements are shown in Additional file 1: Fig. 1, Tables S1-S6. Citrus has non-climacteric fruit ripening (i.e. it ripens without ethylene and respiration bursts), so not surprisingly, no ethylene responsive element (ERE) sites were identified in the citrus promoter sequences, even though ERE is one of the most common elements in other known fruit-specific promoters [40, 41]. Additional file 1 A presents the *cis* elements identified in *CitSEPp*. Analysis detected the presence of a putative TATA-box and a number of CAAT-boxes and a 5′UTR Py-rich stretch, a *cis*-acting element that is associated with high transcription levels. Other interesting elements identified include the CCGTCC-box and CE3, which are responsible for abscisic acid responsiveness (Additional file 1: Fig, 1A, Table S1).

The *CitWAXp* promoter sequence contained several common potential regulatory elements associated with hormone, light and stress responses (Additional file 1: Fig. 1B, Table S2). The presence of these putative *cis* elements indicates that the gene could be regulated by physiological and environmental factors. Putative hormone responsive elements identified in the *CitWAX* promoter region includes an ABRE motif (involved in the abscisic acid responsiveness) and a TCA-element (involved in salicylic acid responsiveness). The *cis*-acting elements involved in light responses includes an ACE motif and four G-box sequences.

*CitUNKp* also contained hormone responsive *cis* elements in the promoter region, including an ABRE motif (involved in the abscisic acid responsiveness) and a TCA-element (involved in salicylic acid responsiveness). Also identified were *cis*-acting elements involved in light responses including an ATCT motif and a G-box motif. In addition, the promoter sequence was found to contain a number of *cis*-elements related to stress responses, including a HSE motif (involved in heat stress responses), an LTR motif (involved in low-temperature responses), a MBS site (MYB binding site involved in drought-induction) and TC-rich repeats (involved in defense and stress responses) (Additional file 1: Fig. 1C, Table S3). The *CitJuSacp* contained potential regulatory *cis* elements associated with hormone, light and stress related responses as well (Additional file 1: Fig. 1D, Table S4). Based on the coding sequence analysis of the *SCAmpPs* genes it’s not surprising that the promoter *CitVO1p* has defense responsive elements such as AT-rich sequence required for minimal elicitor mediated activation in addition to light and hormone responsive elements (Additional file 1: Fig. 1E, Table S5). The presence of these putative *cis* elements indicates that the *CitVO1* gene could also be regulated by physiological and environmental factors. The *PamMybAp* plum promoter was shown to contain putative *cis* elements such as MBSI, MRE and an as-2 box involved in flavonoid and light responsive signaling (Additional file 1: Fig. 1F, Table S6).

### Activity analysis of the promoters using an agroinjection transient assay in tomato fruit

**Fig. 3:**
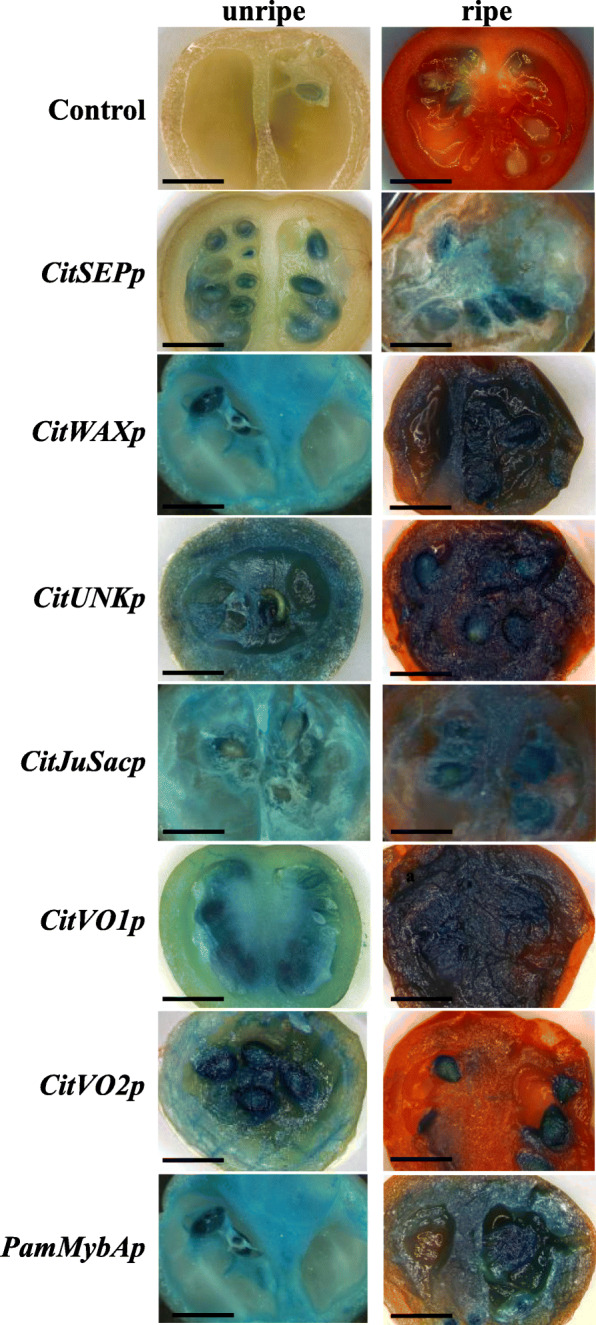
Agroinjection-mediated transient expression in tomato fruit. Results from an *Agrobacterium*-mediated transient expression assay testing the functionality of the candidate fruit-specific promoters in unripe (left) and ripe (right) tomato fruits is shown. Control, wildtype tomato fruit infiltrated with an empty vector is shown on the top, followed below by those infiltrated with the promoter construct as labeled. Each fruit was histochemically stained for β-glucuronidase activity 4 days after agroinjection. The *CitVO2p* construct is a *CitVO1* promoter fragment lacking the native intron. Scale bar – 1 cm

The ability of the candidate promoters to confer gene expression was first investigated using *Agrobacterium*-mediated transient expression assay in Micro-Tom tomato fruits. Fruits at immature green and red ripe stages (22–25 d after anthesis) were infiltrated using *Agrobacterium* suspensions containing the constructs shown in (Fig. 2). β-glucuronidase activity was absent in tomato fruits infiltrated with the empty vector control, whereas strong or varied expression patterns were detected in fruits injected with various candidate promoter constructs (Fig. 3). The different candidate promoters showed different intensities of GUSPlus staining in immature versus ripe fruit 4 d after infiltration. The *CitSEPp* injected fruit showed expression in immature and ripe fruits; the staining was appeared mostly in the mucosal sack around the seeds and the seed itself. Samples injected with the *CitWAXp*, *CitJuSacp* and *PamMybAp* constructs showed strong expression in most tissues of both immature and ripe fruit (including the central lamella, placental and pericarp tissues), but staining was not detected in the outer epidermal layer. *CitUNKp* injected fruits produced the strongest GUSPlus staining in both young immature tomato fruits as well as in mature ripe fruits throughout all tissues except the outer epidermal layer. *CitVO1p* infiltrated fruit exhibited expression that was stronger in mature ripe tomatoes than young fruits, while *CitVO2p* (a version of the *CitVO1* promoter lacking the intron) staining was predominantly visible only in the seeds of the injected fruits. Taken together, these observations demonstrated that the candidate promoters successfully conferred expression of the *GUSPlus* reporter gene in tomato fruit tissue via agroinjection, indicating that the promoter candidates from citrus and plum contain active promoter elements that are functional in tomato fruit tissue. Based on the observed results, *CitSEPp*, *CitWAXp*, *CitJuSacp*, *PamMybAp*, *CitUNKp* and *CitVO1p* were chosen for further study in stably transformed tomato plants.

### Characterization of stable transgenic micro-tom tomato lines

**Fig. 4:**
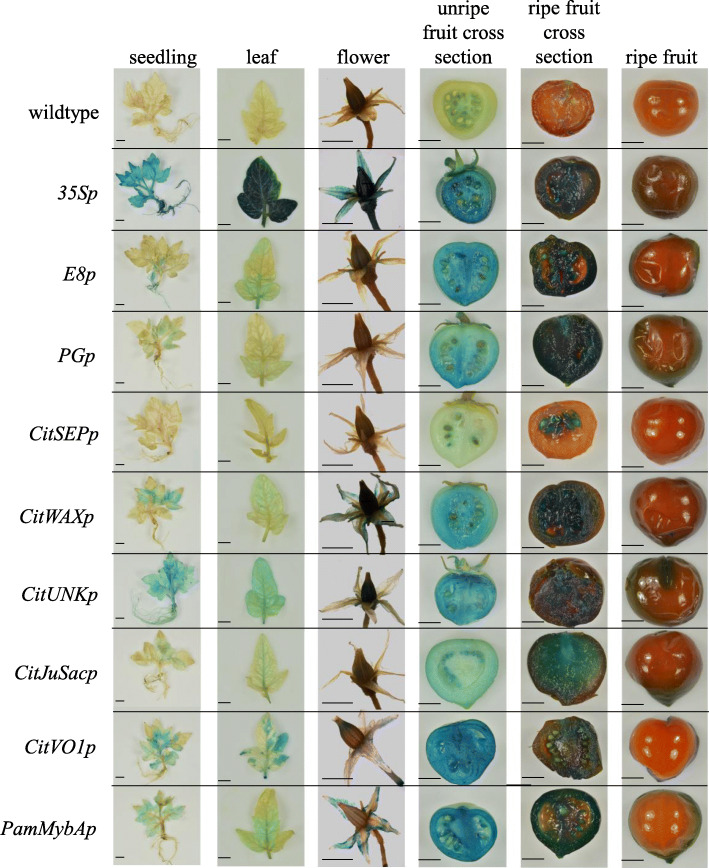
Histochemical staining of vegetative and fruit tissues from wildtype and transgenic tomato lines. Histochemical staining of whole seedlings mature leaves, flowers, cross sections of unripe and ripe fruit, and whole unripe fruit are shown. Each row contains representative images from wildtype or a corresponding promoter-GUSPlus transgenic line. Scale bar – 1 cm

**Fig. 5:**
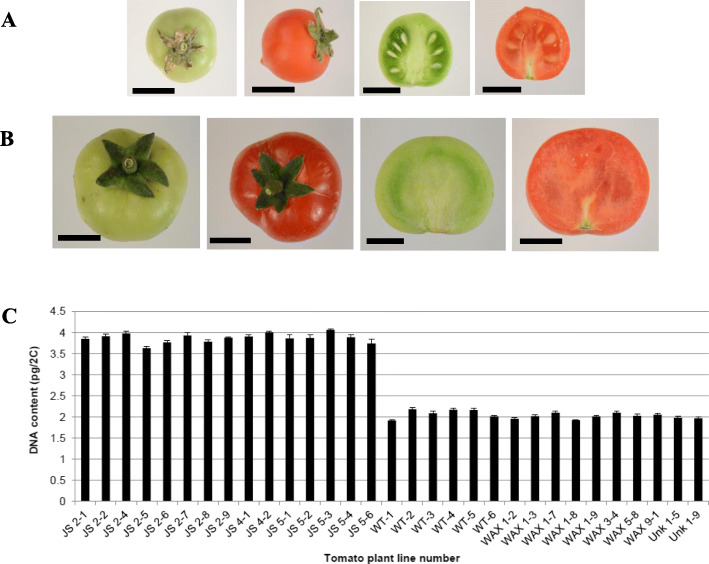
Seedless phenotype in transgenic CitJuSacp-GUSPlus tomato lines. **a** Wildtype and **b** *CitJuSacp* transgenic green and ripe fruit. **c** The measured genomic DNA content (picograms/2C) in *CitJuSacp* (JS), *CitWAXp* (WAX) and *CitUNKp* (UNK) transgenic lines and Micro-Tom wildtype (WT) lines. Scale bar =1 cm

A total of 15–20 independent transgenic tomato lines were generated for each promoter construct using *Agrobacterium*-mediated transformation and were grown in a greenhouse (See Methods). The individual lines were grown to maturity, with their overall growth and development monitored. They were validated using genomic PCR to confirm the presence of the *nptII* selection marker sequence. Compared to wildtype, the transgenic plants showed no significant differences in either vegetative or reproductive growth patterns (Fig. 4), with the exception of the *CitJuSacp* transgenic events (Fig. 5, discussed below). Based on initial reporter gene analyses on vegetative and reproductive tissues, T_1_ seeds were collected from selected T_0_ transgenic lines.

### Analysis of the fruit specific/preferential promoters in transgenic tomato

Qualitative analysis of promoter activity was conducted by comparing the intensity of GUSPlus histochemical staining between promoter transgenic lines and controls. Images showing GUSPlus staining in various vegetative and reproductive tissues are presented in Fig. 4. As expected, wildtype plants did not display any GUS staining in either vegetative or reproductive tissues, while the positive control transgenic lines transformed with CaMV*35S* promoter fused to reporter gene showed GUSPlus staining in all of the tested tissues. The control fruit-specific tomato *E8p*-*GUSPlus* lines showed strong activity in both unripe and ripe fruits, weak activity in flowers, seedlings and mature leaf samples as previously observed by others [18–21]. The fruit-specific *PGp* promoter lines also exhibited weak GUSPlus staining in seedlings, but no activity in mature leaves, roots or flowers. The GUSPlus staining in *PGp* promoter lines was very strong in ripe fruit compared to that in young immature fruit, consistent with PG’s involvement in the fruit softening process (Fig. 4). *CitSEPp* transgenic lines showed no detectable GUS expression in petiole, root, flower, or mature leaf and only faint GUSPlus staining in young immature fruit but exhibited strong straining in tissues around the seeds of ripe fruit. The staining in the fruit appeared strongest in the mucosal sac surrounding the seeds, followed by locular tissue, pericarp tissue and placental tissue (Fig. 4). *CitWAXp* lines showed some weak/variable GUSPlus staining in seedling leaf and flower tissues, but little or no expression was observed in the root and mature leaf samples, and strong staining occurred in both immature and ripe fruits. *CitUNKp* lines showed mild GUSPlus staining in seedling, mature leaf, stem, root, and flower, and strong expression in immature and ripe fruits. *CitJuSacp* lines showed some faint GUSPlus staining in seedling tissues and the flower, but no staining in mature leaves and roots. These lines also exhibited weak to moderate strength staining in unripe fruit and strong staining in ripe fruit (Fig. 4). *CitVO1p* lines showed weak staining in leaves, flowers, and stem with the strong staining at abscission zones and at wound sites. The immature as well as ripe fruits showed the darkest/most robust staining (Fig. 4). *PamMybAp* transgenic lines showed a unique pattern of GUSPlus staining in leaves with blue precipitate forming near the mid-rib and the base of the leaf, while the root did not show any staining. The flower had blue stained petals, but not sepals and both unripe and ripe fruits of the *PamMybAp* lines exhibited very strong GUSPlus staining (Fig. 4).

Seed and fruit development are intimately related processes controlled by internal signals and environmental cues. Interestingly, the *CitJuSacp* transgenic lines generated seedless fruits, even though the overall fruit development timing was similar to that of wildtype (Fig. 5). The *CitJuSacp* lines’ fruit were noticeably larger in size compared to wildtype and either did not develop seeds or developed small non-viable seeds (Fig. 5a, b). The DNA content (or Cot value) of the seedless *CitJuSacp* tomato transgenic events indicated that surprisingly, all of the tested lines had double the DNA content compared to wildtype Micro-Tom. The tested *CitWAXp* and *CitUNKp* lines generated under identical tissue culture conditions contained the wildtype amount of genomic DNA (Fig. 5c). These results suggest that the presence of the *CitJuSacp* T-DNA correlated strongly with a genome duplication event. The seedless nature of the *CitJuSacp* tomato lines is a phenomenon specifically associated with the *CitJuSacp* construct and the tomato genome, since transformation of the same construct into *Arabidopsis* did not affect fertility (data not shown). The *CitJuSacp* transgenic Micro-Tom lines exhibited neither a noticeable loss in the quality, nor productivity, and the plants did not display vegetative or reproductive alterations beyond the lack of producing viable T_1_ seeds.

Droplet digital PCR (ddPCR) was conducted to measure the transgene copy number of all lines in the T_1_ generation (Additional file 2: Table S1, S2) as previously described [42]. ddPCR results of the T_0_ *CitJuSacp* lines had a ‘half’ copy (due to genome doubling, this is 1 copy per tetraploid genome) or single copy (2 copies per tetraploid genome) of the *nptII* transgene. The ‘half-copy’ plants occasionally produced non-viable seeds and the ‘single copy’ lines produced no visible seed (Fig. 5b). These results are consistent with the genomic DNA content analyses that showed that the *CitJuSacp* transgenic events contained twice the amount of DNA than wildtype Micro-Tom plants (Fig. 5c) and verified that the recovered transgenic lines had undergone a complete genome duplication.

**Fig. 6:**
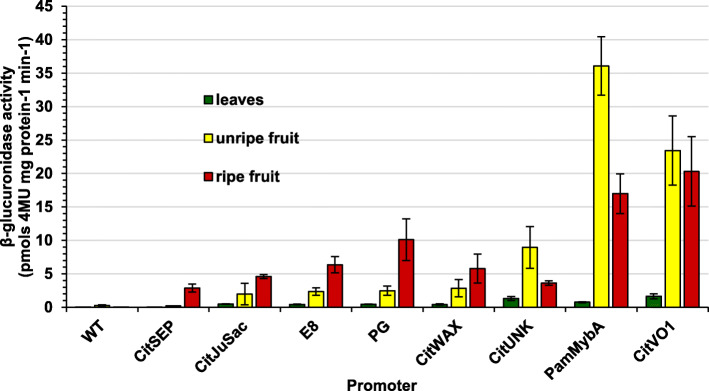
β-glucuronidase activity in measured in tomato fruit and leaves. The measured β-glucuronidase activity in leaves (green bars), unripe fruit (yellow bars) and ripe fruit (red bars) from wildtype (WT) and representative transgenic tomato lines is shown. The mean of nine samples (except *CsJuSacp* with *n* = 6), with their standard error bars (the error bars) are shown

To analyze the specificity of promoter observations, β-glucuronidase activity was measured in leaves, immature fruit, and ripe fruit from three representative transgenic tomato lines for each promoter construct (Fig. 6 and Additional file 4: Table S1). As expected, wildtype plants contained little or no detectable β-glucuronidase activity. Interestingly, the *CitSEPp* transgenic lines showed GUSPlus activity only in ripe fruits with only background activity in leaves and unripe fruits (Fig. 6 and Additional file 4: Table S1). These results are consistent with the results gathered from the agroinjection transient expression assays as well as the histochemical staining analyses (Figs. 3 and 4). *CitWAXp* and *CitJuSacp* transgenic lines showed activity similar to *E8p* and *PGp* positive control lines in both unripe and ripe fruit, while *CitVO1p* and *PamMybAp* transgenic lines showed higher activity levels (Fig. 6). *CitUNKp* and *PamMybAp* were unique in that they showed significantly higher expression in unripe fruit, but lower expression in ripe fruit when compared to the *E8p* and *PGp* controls (Fig. 6 and Additional file 4: Table S1). To further assess promoter activity levels, statistical analyses were done to compare all promoters in each tissue. Using Kruskal-Wallis rank sum tests, significant differences between promoters were seen for leaves (χ^2^ = 51.55, df = 8, *p* = 2.055 × 10^− 8^), unripe fruits (χ^2^ = 57.65, df = 8, *p* = 1.344 × 10^− 9^), and ripe fruits (χ^2^ = 51.198, df = 8, *p* = 2.404 × 10^− 8^). Wilcoxon post-hoc tests were performed for all pair-wise comparisons with results presented (Additional file 4: Table S2).

Based on the above results, the citrus candidate promoter *CitSEPp* had the tightest fruit-specific expression pattern in tomato. To further confirm the fruit-specific pattern of *CitSEPp*, transgenic *Arabidopsis* and tobacco transgenic lines were also generated and characterized. Histochemical staining of T_1_ transgenic lines of *Arabidopsis* seedlings and tobacco leaf and flower samples did not exhibit any detectable staining. However, β-glucuronidase activity was detected in the seeds of the transgenic *Arabidopsis* lines and in the stigma and ovary of the transgenic tobacco lines (Additional file 3: Figs. 1, 2). These results demonstrate that *CitSEPp* confers a highly specific expression pattern in tomato, *Arabidopsis* and tobacco, albeit a somewhat distinct pattern in each species.

Taken together our results demonstrate that *CitWAXp*, *CitJuSacp*, *CitVO1p*, *CitUNKp* and *PamMybAp* are strong fruit-preferential promoters and *CitSEPp* is a tightly controlled fruit/seed-specific promoter for fruit biotechnology based on observed GUSPlus expression patterns and the intensity of staining observed in both transient and stable transgenic tomato tissues.

## Discussion

In this study, five different citrus promoters and one plum promoter were evaluated in transgenic tomato. A search for promoter *cis* elements revealed the presence of many putative regulatory motifs in each of the promoters. Analysis determined the presence of a TATA box and several CAAT boxes that are conserved among all promoter sequences (Additional file 1: Fig. S1). Sequences that were responsive to hormones, an anaerobic responsive element, GATA boxes, pyrimidine box and other *cis* elements that confer fruit or organ expression specificity in other promoters were found in the citrus and plum promoters. In addition, light responsive *cis* elements such as ACE, SP1 G-box and MRE sequences were identified in the *CitSEP*, *CitWAX*, *CitJuSac*, *CitVO1* and *PamMybA* promoter sequences. The presence of these *cis* elements indicates an interaction of plant and environmental factors during the process of fruit ripening. The *CitUNK* promoter region included a number of cold, drought and heat-responsive elements and exhibited among the highest expression in unripe fruit, leaf and seedling tissues, compared to the other candidate promoters.

Promoters from potato, pepper, peach, strawberry, melon and apple showed pronounced activity in tomato, usually similar to the gene expression pattern detected in the source plant [25–27, 43–46]. Our results add to the evidence supporting that tomato is a useful model system for heterologous promoter testing, especially when promoters with fruit expression specificity are being analyzed. Our results also demonstrate that agroinjection-mediated transient expression assays carried out in Micro-Tom tomato fruit are predictive of the expression observed in stably transformed tomato plants (Figs. 3 and 4), suggesting that this approach can be used as a rapid assay to assess candidate fruit-specific promoters from citrus and potentially other species. Overall, our results also indicate that the candidate citrus fruit promoters functioned in transgenic tomato in line with the expectations based on the native genes’ expression in citrus, in terms of both tissue localization and the quantitative differences in expression between the candidates.

*CitSEPp* lines exhibited the lowest expression levels, but also the highest tissue specificity among the five citrus promoters analyzed. This promoter had the tightest control seen for all five analyzed activating expression in ripe fruit, without detectable expression in leaves or other vegetative organs. The activity of the *CitWAXp* and *CitJuSacp* promoters exhibited strong fruit preferential expression with weak expression in seedlings and young leaves. This pattern is similar to that observed for the fruit-specific tomato *E8* and *PG* promoters [18]. In addition, the *CitWAXp* lines were found to have moderate levels of activity in flowers (Fig. 6 and Additional file 4: Table S2). It was also found that the *CitJuSacp* transgenic lines exhibited fruit-preferential expression, consistent with prior published results for the orthologous *Cl111* promoter from lemon [37] but surprisingly, this construct also consistently generated tetrapoid transgenic Micro-Tom plants carrying only 1 or 2 transgene copies and produced seedless tomato fruit (Fig. 5 and Additional file 2: Table S1). The mechanism by which transformation with the *CitJuSacp* construct stimulates genome duplication is unknown, although results suggest that genome duplication was a selected event in the transformation process due to an effect of the transgene construct on plant implicating a possible negative effect on development. This speculation will require further research in the *CitJuSacp* transgenic lines to verify.

The *CitVO1* and *PamMybA* promoters showed quantitatively high levels of *GUSPlus* activity in immature and mature fruit tissues, compared to the other tested promoters (Figs. 3, 4 and 6 Additional file 4: Table S2). However, *CitVO1p* and *PamMybAp* also had low, but detectable levels of activity in most of the tested vegetative tissues as well. *CitVO1p* contains defense-related *cis* elements and an AT-rich sequence element for elicitor-mediated activation, consistent with the hypothesis that the native gene may be expressed as part of an active defense response (Additional file 1: Table S5). β-glucuronidase activity was also detected in the abscission zone and tissues injured during sample processing for the *CitVO1p* transgenic lines. Overall, the levels of expression conferred by the candidate promoters were generally comparable to those of the tomato fruit-specific *E8p* and *PGp* constructs (Figs. 4 and 6 and Additional file 4: Table S2).

Gene transfer technology is enabling the metabolic engineering of plants, providing a better understanding of the regulation of complex metabolic processes allowing the tailoring of these pathways to meet our needs [47]. The promoters we describe, are confirmed to be fruit specific/preferential and can be used in the future for fruit metabolic engineering in tomato and potentially other species. While regulatory and limited social acceptance hinder the commercial development of transgenic fruit crops alternative genetic engineering approaches may offer a solution. Approaches such as cisgenesis or intragenesis that utilize native plant sequences for genetic engineering are receiving increased interest and may require less regulatory scrutiny and increased public acceptance [48]. These types of approaches have been implemented by Joshi et al., [49] showing that the *HcrVf1* and *HcrVf2* genes, together with their native promoters and terminators (cisgenic approach) or combined with regulatory sequences of the apple *rubisco* gene (intragenic approach) conferred resistance to apple scab. The cisgenic approach has also been used to develop other disease-resistant apple [48] and grapevine [50] cultivars. In other studies, tomato fruits with enhanced rot resistance and improved shelf life were obtained by expressing a tomato anionic peroxidase under control of a fruit-specific *E8* promoter [51]. The promoters identified and characterized here can potentially be used to engineer citrus fruit in an intragenic manner providing enhanced nutritional content by over-accumulating antioxidants or other desirable gene products. For example, the promoters identified in this study could be used to control fruit-preferential expression of *MoroMybA* gene [39] to elicit anthocyanin expression in citrus fruit, to develop novel blood orange-like cultivars [52]. This would provide cultivars with consistent fruit color without the requirement for specific environmental conditions currently required to produce blood orange [53].

## Conclusion

In summary, all of the candidate promoters (except *CitSEPp)*, were found to confer fruit preferential expression, with their highest expression levels in fruit tissues including the pericarp, placenta, locule and columella, but they also exhibited weak activity in various vegetative or reproductive tissues. The exception was the *CitSEP* promoter that exhibited fruit/seed-specific expression only in ripe tomato fruit. The *CitWAX* and *CitJuSac* promoters exhibited expression activities that were similar to the patterns observed for the tomato fruit ripening-specific promoters *E8* and *PG* [19, 21] while *CitVO1p* and *PamMybAp* exhibited the strongest fruit expression but with a subsequent increase in vegetative activity. The *CitUNK* and *PamMybAp* promoters were unique in that the strongest expression was seen in unripe instead of ripe tomato fruit. Its hoped that these novel fruit-specific/preferential promoters will be useful as molecular tools for plant research as well as the engineering of novel traits in fruit crops in the near future.

## Methods

### Plant material and promoter isolation

Sweet orange (*Citrus sinensis*) plant leaves were used to isolate the citrus promoters. Citrus genomic DNA was isolated from 5 g of young leaves obtained from purchased trees (Eastbay Nurseries, Berkeley CA). Leaves were frozen in liquid nitrogen and the tissue was ground to fine powder from which genomic DNA was isolated. The Gentra Puregene DNA Purification Kit (Qiagen) was used following the manufacturer’s extraction protocol for ‘Frozen Leaf Tissue’. Primers used for PCR amplification of the citrus promoter sequences are listed in Table 2. The Plum promoter *PamMybAp* was isolated from young leaves of the wild plum variety *Prunus Americana* trees obtained in El Cerrito CA [39]. Leaves were grounded in liquid nitrogen, from which genomic DNA was extracted using the EZNATM High Performance (HP) DNA Kit (Omega Bio-Tek) with the addition of 2% Polyvinylpyrrolidone-40 (PVP-40) (w/v) to CPL buffer and 2-mercaptoethanol. Genomic DNA quantity was assessed using the Quant-iT PicoGreen kit (Invitrogen). A total of 2 μg of purified DNA was provided to David H Murdock Research Institute, Kannapolis, NC for library construction and sequencing. A paired-end and a mate-pair library were constructed with an average insert size of 375 bp and 2950 bp, respectively. These libraries were sequenced using an Illumina HiSeq 2000 sequencer. A total of 194,856,870,100-bp paired-end sequence reads and 158,319,386 mate-pair sequence reads were obtained. Sequence reads were assembled against the peach (*Prunus persica*) genome version 2 [54], (https://www.rosaceae.org/species/prunus_persica/genome_v2.0.a1) using the CLC Genomics Workbench version 8.5 assembly tool [55] (CLC bio/Qiagen) with two modifications to the default settings: length fraction = 0.7 and similarity fraction = 0.85. The ~ 2000 bp assembled sequence upstream of the known *Prunus domestica MybA* gene was predicted to be the promoter of the gene. Primers were designed based on the assembled *Prunus domestica* genomic sequence (with the reverse primer positioned inside the open reading frame of *MybA*) to amplify across the predicted region for isolation of the candidate plum promoter *PamMybAp* from the wild plum *Prunus americana* (Table 2). Q5 high fidelity DNA polymerase (New England Biolabs) was used for promoter amplification following recommended conditions using genomic DNA as a template. Putative promoter amplicons were cloned (see below) and sequence confirmed.

### Plasmid construction

The various promoter regions were PCR amplified using Q5 high fidelity DNA polymerase (NEB) promoter specific primer pairs and cloned into a modified pCTAGII binary vector to control *GUSPlus* reporter gene expression. The pCTAGII-GUSPlus vector (GenBank accession MG818373) has been used for all the promoter cloning including plum and tomato control promoters. Tomato promoter *E8p* and *PGp* were isolated from (*Solanum lycopersicum* cv VF3) using promoter specific primers (5′-tttgaattcATTTTTGACATCCCTAATGATATTG-3′ and 5′-tttccatggCTTCTTTTGCACTGTGAATGATTAG-3**′**) for E8 promoter and (5′-tttcctgcagggcttcttaaaaaggcaaattgattaatttg-3′ and 5′-tttccatgggatatattgttatatggtatggtttttaaac-3′) for PG promoter and cloned into pCTAGII-GUSPlus binary vector. *Solanum lycopersicum* cv VF3 genomic DNA was obtained from Dr. Shelia McCormik at the Plant Gene Expression Center/UC Berkeley, Albany CA. Molecular constructs that contain the candidate promoters were confirmed using DNA sequencing of plasmid DNA isolated using the ZR plasmid miniprep-classic kit (Zymo Research Corp.) following the manufacturer’s instructions.

### Promoter sequence analysis

Analysis of putative *cis*-regulatory elements within the citrus promoter was performed with the Plant Promoter Analysis Navigator, the Plant Cis Acting Regulatory Element (PlantCARE) search tool [40] and the Database of Plant Cis acting Regulatory DNA Elements. Additional known *cis* elements that were not included within the above websites’ databases were queried and annotated manually. Websites of the tools used in this study are: http://plantpan.mbc.nctu.edu.tw/index.php [56]; http://bioinformatics.psb.ugent.be/webtools/plantcare/html/ [57]; http://www.dna.affrc.go.jp/PLACE/ [58];

http://133.66.216.33/ppdb/cgi-bin/index.cgi [59];

https://www.rosaceae.org/species/prunus_persica/genome_v2.0.a1 [54];

http://citrus.hzau.edu.cn/cgi-bin/blast/blast.cgi [60];

www.citrusgenomedb.org/;

http://citrus.pw.usda.gov/;

http://www.phytozome.net/citrus.php [61];

www.ncbi.nlm.nih.gov/geo [62];

### *Agrobacterium*-mediated transient expression

All binary vectors were transformed into *Agrobacterium tumefaciens* strain GV3101 and selected on LB plates supplemented with kanamycin and gentamycin at 100 mg/L. *Agrobacterium* cultures (5 mL) were grown overnight from individual colonies at 28 °C in LB medium plus selective antibiotics, transferred to 50 mL of induction medium (0.5% beef extract, 0.1% yeast extract, 0.5% peptone, 0.5% sucrose, 2 mM MgSO_4_, 20 mM acetosyringone, 10 mM MES, pH 5.6) plus antibiotics, and grown again overnight. Next day, cultures were recovered by centrifugation, resuspended in infiltration medium (10 mM MgCl_2_, 10 mM MES, 200 mM acetosyringone, pH 5.6; optical density at 600 nm of 1.0), and incubated at room temperature with gentle agitation (20 rpm) for a minimum of 2 h. Cultures were then syringe injected into three unripe or ripe tomato fruits (*Solanum lycopersicum* cv Micro-Tom *Rg1*) of similar age and size for each construct. A 1 mL syringe with a 0.5–316 mm needle (BD Pastipak) was used by inserting the needle 3 to 4 mm in depth into the fruit the stylar apex, and gently injecting approximately 600 μl of the *Agrobacterium* solution. The progress of the injection process could be followed by a slight darkening of the infiltrated areas. Once the entire fruit has been infiltrated, some drops of infiltration solution would begin to exude from the hydathodes at the tip of the sepals. Only completely infiltrated fruits were used in the experiments. An *Agrobacterium* strain carrying the empty pCTAGII-GUSPlus vector was used as a negative control in the assay.

### *Agrobacterium*-mediated stable tomato transformation

Transgenic Micro-Tom *Rg1* tomato plants were transformed with the citrus, plum and tomato promoters using *Agrobacterium tumefaciens* strain GV3101 carrying the pCTAGII-GUSPlus derived binary vector constructs and kanamycin selection as described in [63, 64]. Seed for Micro-Tom *Rg1* tomato transformation was obtained from Dr. Peres, Brazil. In brief, *Agrobacterium* cells were grown to an optical density of 1.0 at 600 nm (OD_600_), and a final suspension at OD_600_ of 0.5 was used for cocultivation. Young, healthy green leaves were cut into pieces approximately 10 mm in length, and the leaf segments were incubated in an *Agrobacterium* suspension for 30 min. The leaf segments were then blotted dry on sterile filter paper for 5 min and then placed onto MS co-cultivation medium (Sigma) in sterile Petri dishes and kept in the growth chamber at 25 °C for 3 days in the dark. The infected leaf explants were then transferred to regeneration/selection medium and incubated at 24 °C with 16 h of light and 8 h of dark). After 2–3 weeks, the leaf explants were transferred onto fresh regeneration/selection media. Regenerated shoots from explants were excised carefully and transferred into plant culture dishes containing rooting medium. Rooted plants were transferred to Sunshine potting mix (Sun Gro Horticulture Ltd.) and grown in the greenhouse with 16 h of light at 150 photosynthetic photon flux density (μmol photons m^2^ s^1^) at 23 °C and 8 h of dark at 20 °C with 70% humidity. A total of 20–25 kanamycin resistant lines were obtained from the T_0_ generation for each test promoter construct. T_1_ seeds were collected and selected on an MS plate supplemented with kanamycin 100 mg/ml, PCR verified for the transgene and used for further analysis as described below.

### Genomic PCR

Genomic DNA was extracted by maceration of a 1 cm^2^ piece of leaf tissue in 400 μL of buffer (200 mM Tris–HCl pH 7.8, 250 mM NaCl, 25 mM EDTA, 0.5% SDS). After centrifugation and isopropanol precipitation, the pellet was washed with 70% ethanol and resuspended in 50 μL of water with 1 mM RNase A. PCR amplification was performed using 2 μL of genomic DNA in reactions with a total volume of 25 μL. Presence of the transgene was confirmed by PCR using transgene specific primers (*nptII* For 5′- TTGCCGAATATCATGGTGGA 3′ and *nptII* Rev. 5′ TCAGCAATATCACGGGTAGC- 3′). Droplet digital PCR (ddPCR) was performed following the methods previously described on T_1_ siblings [42].

### Qualitative and quantitative GUSPlus analysis

β-glucuronidase activity was detected using a GUS staining solution (0.1 M sodium phosphate pH 7.0, 0.5 mM potassium ferrocyanide, 0.5 mM potassium ferricyanide, 1.5 g/L X-gluc, and 0.5% v/v Triton X-100) generally for 4 h to 20 h at 37 °C. The incubation time was adjusted based on the strength of the staining observed. After staining, green tissues were passed through several changes of 70 and 95% ethanol to remove chlorophyll and then used for imaging.

The activity of β-glucuronidase (GUSPlus) was quantified in extracts of plant tissue using 4-methylumbelliferyl β-D-glucuronide (4-MUG) as a substrate (Gold Biotechnology). Using excitation at 365 nm and measuring emission at 455 nm, the amount of 4-MU produced in the assay was quantified as described in [65–67]. Nine replica samples were used to determine GUS activity. Nine replica samples were used to determine GUS activity for all promoters, except *CitJuSacp* where *n* = 6. Statistical analyses were done in R (version 3.6.3). Visual inspections of data were performed with boxplots and residual plots, and Shapiro-Wilk normality tests were done to inspect ANOVA assumptions. Criteria for ANOVA were not met. Thus, Kruskal Wallis rank sum tests were done, followed by post-hoc Wilcoxon tests (with Benjamini Hochberg adjustment) for all pair-wise comparisons, using kruskal.test() and pairwise.wilcox.test() in R.

### Imaging of transgenic plants

The photographs of the plants and fruits were recorded using a Nikon D7000 digital camera with an AF Micro Nikkor 60 mm 1:2.8 D lens or AF-S Nikkor 18–70 mm DX lens (Nikon Inc.) under tungsten lamps (Philips, 120 V, 300 W). The camera was set manually for all parameters including ISO sensitivity, focus, f-stop and time. A photography gray card was used as a reference to get the correct exposure. The agroinjection images and GUSPlus stained tomato fruits were observed and photographed in a Leica MZ16-F (Leica Microsystems, Inc.) stereo zoom light stereoscope equipped with a QImaging Retiga 2000 R fast cooled, digital color camera.

### Cot value DNA analysis

Fresh leaves of various transgenic tomato lines were sent to Benaroya Research Institute, WA for cot value analysis. Total DNA content was measured in picograms/2C. The procedure used to analyze nuclear DNA content in plant cells was modified from Arumuganathan and Earle [68]. Briefly, intact nuclei were prepared by chopping of 50 mg plant tissues in MgSO_4_ buffer mixed with DNA standards and stained with propidium iodide (PI) in a solution containing DNAase-free-RNAase. Fluorescence intensities of the stained nuclei were measured by a FACS caliber flow cytometer (Becton-Dickinson). Values for nuclear DNA content was estimated by comparing fluorescence intensities of the nuclei of the test population with those of an appropriate internal DNA standard that is included with the tissue being tested. Nuclei from *Arabidopsis thaliana* (0.36 pg/2 C) used as internal standard. For each measurement, the propidium iodide fluorescence area signals (FL2-A) from 1000 nuclei were collected and analyzed by CellQuest software (Becton-Dickinson). The mean position of the G0/G1 nuclei peak of the sample and the internal standard were determined by CellQuest software. The mean nuclear DNA content of each plant sample, measured in picograms, were based on 1000 scanned nuclei.

## Supplementary information

**Additional file 1: Figure S1.** Schematic representation of the promoter region. The various *cis* elements identified in the candidate promoters *CitSEPp*, *CitWAXp*, *CitUNKp*, *CitJuSacp*, *CitVO1p* and Plum *PamMybAp* are shown as colored bars, respectively. The entire nucleotide sequences are available from the GenBank under the accession numbers *CitSEPp* (MK012379), *CitWAXp* (MK012380), *CitUNKp* (MK012381), *CitJuSacp* (MK012382), *CitVO1p* (MK012383), *CitVO2p* (MK012384), *PamMybAp* (MK012380). **Table S1.** Promoter elements identified in *CitSEPp.* **Table S2.** Promoter elements identified in *CitWAXp.* **Table S3.** Promoter elements identified in *CitUNKp.* **Table S4.** Promoter elements identified in *CitJuSacp.* **Table S5.** Promoter element identified in *CitVO1/2p.* **Table S6.** Promoter elements identified in *PamMybAp.*

**Additional file 2: Table S1.** Transgene copy number measurements in T_1_ sibling transgenic Micro-Tom tomato lines from 3 independent events (4 plants from each family). **Table S2.** ddPCR primers and probe for reference and transgene detection.

**Additional file 3: Figure S1.** Histochemical staining of *CitSEPp* controlled GUSPlus activity in transgenic *Arabidopsis* tissues. (A) A whole seedling, (B) flowering inflorescence and (C) a developing silique stained for β-glucuronidase activity are shown. **Figure S2.** Histochemical staining of *CitSEPp* controlled GUSPlus activity in transgenic tobacco. Representative images for *CitSEPp* T_0_ transgenic tobacco (*Nicotiana tabacum* L. cv. Petit Havana SR1) tissues stained for β-glucuronidase activity are shown. (A) young leaf, (B) mature leaf (C) flowers with detectable staining in stigma and the flower base (ovule), circled. (D) stigma (E) bisected ovule

**Additional file 4: Table S1.** β-glucuronidase activity in measured in tomato fruit and leaves. The mean of nine samples (except *CsJuSacp* with *n* = 6), with their standard error bars (the error bars) are shown (see Fig. 6). **Table S2.** β-glucuronidase activity in measured in tomato fruit and leaves. The measured β-glucuronidase activity in leaves (green), unripe fruit (yellow) and ripe fruit (red) from wildtype (WT) and representative transgenic tomato lines is shown from the mean of nine samples (except *CsJuSacp* with *n* = 6). The median and interquartile range are indicated in boxplots. Kruskal-Wallis rank sum tests indicated significant differences between promoters for leaves (χ^2^ = 51.55, df = 8, *p* = 2.055 × 10^− 8^), unripe fruits (χ^2^ = 57.65, df = 8, *p* = 1.344 × 10^− 9^), and ripe fruits (χ^2^ = 51.198, df = 8, *p* = 2.404 × 10^− 8^). Post-hoc Wilcoxon tests were performed for all pair-wise comparisons.

## Data Availability

The datasets used to predict potential tissue specific promoters during the study are available from the corresponding author on reasonable request. Web sites from which the data was generated is listed within the manuscript. All data generated or analysed during this study are included in this published article and its supplementary information files. Nucleotide sequences are available from GenBank under the accession numbers *CitSEPp* (MK012379), *CitWAXp* (MK012380), *CitUNKp* (MK012381), *CitJuSacp* (MK012382), *CitVO1p* (MK012383), *CitVO2p* (MK012384), *PamMybAp* (MK012380).

## Abbreviations

ACE: ACGT sequence containing light responsive *cis* element of a promoter
ddPCR: Droplet digital PCR
EST: Expressed Sequence Tag
G-box: A CACGTG sequence containing *cis* element of a promoter
GEO: Gene Expression Omnibus
*HcrVf1* and HcrVf2: Cf resistance gene homologs that are present in the Vf region. These genes are homologs to *C. fulvum* resistance genes of the Vf region
HLB: Huanglongbing
HSE: Motif or type of promoter *cis* element involved in heat stress responses
LTR: Motif or type of promoter *cis* element involved in low-temperature responses
MRE: CAACTG sequence containing MYB binding site *cis* element involved in drought-inducibility and light responsiveness
MUG: Methylumbelliferyl β-D-glucuronide
PlantCARE: Plant Cis Acting Regulatory Element
*Rg1*: Regeneration1 *allele*
SP1: GCCCCGCCCA sequence containing *cis* element of a promoter
