# Supplementary material for: Isolation of novel citrus and plum fruit promoters and their functional characterization for fruit biotechnology
Source: BMC Biotechnol. 2020 Aug 20;20:43. doi: 10.1186/s12896-020-00635-w (PMC7439555; doi:10.1186/s12896-020-00635-w)
Supplement: Supplementary file 1 — Additional file 1: Figure S1. Schematic representation of the promoter region. The various cis elements identified in the candidate promoters CitSEPp, CitWAXp, CitUNKp, CitJuSacp, CitVO1p and Plum PamMybAp are shown as colored bars, respectively. The entire nucleotide sequences are available from the GenBank under the accession numbers CitSEPp (MK012379), CitWAXp (MK012380), CitUNKp (MK012381), CitJuSacp (MK012382), CitVO1p (MK012383), CitVO2p (MK012384), PamMybAp (MK012380). Table S1. Promoter elements identified in CitSEPp. Table S2. Promoter elements identified in CitWAXp. Table S3. Promoter elements identified in CitUNKp. Table S4. Promoter elements identified in CitJuSacp. Table S5. Promoter element identified in CitVO1/2p. Table S6. Promoter elements identified in PamMybAp. [file 12896_2020_635_MOESM1_ESM.pptx]

## Slide 1
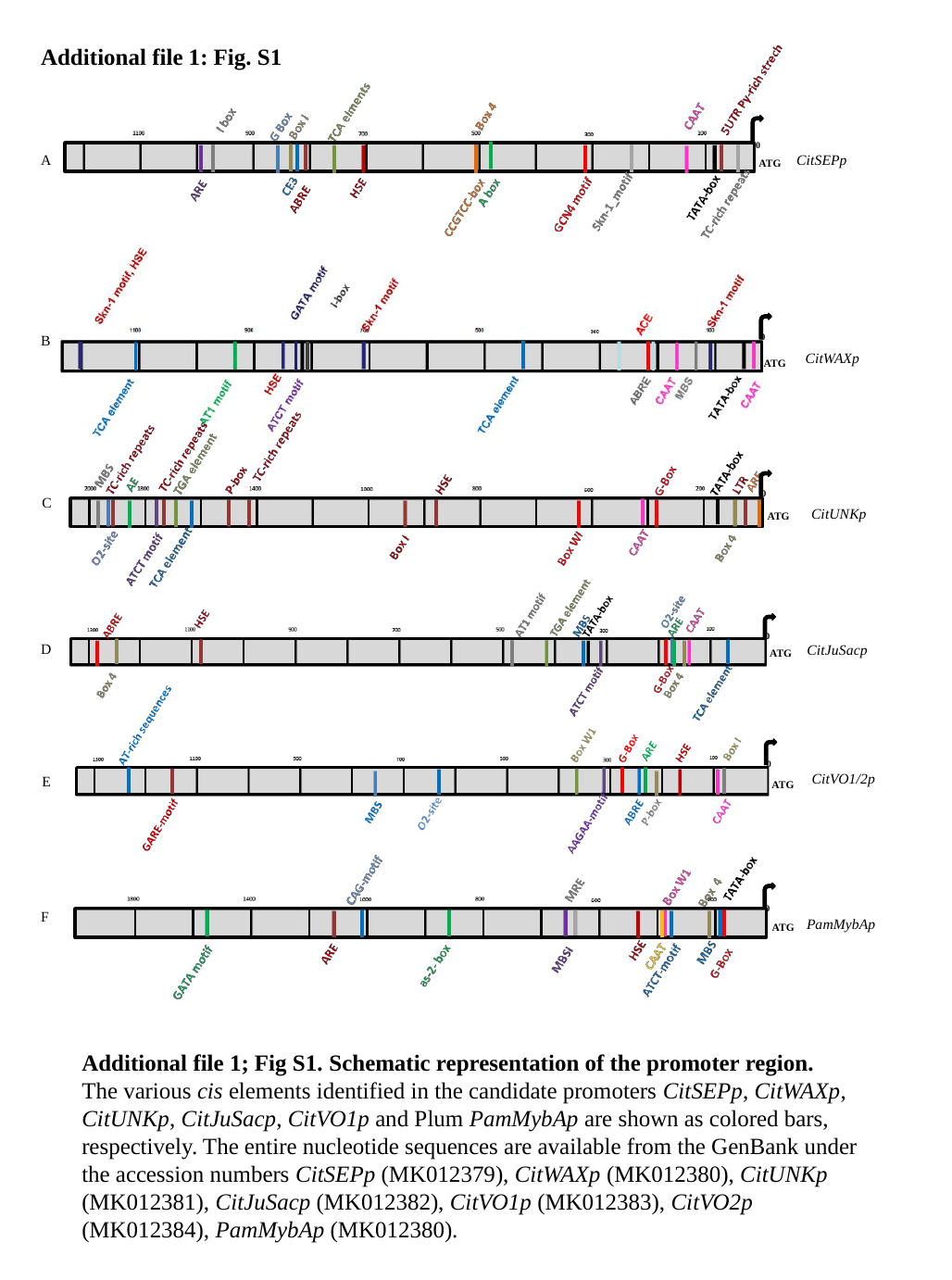

CitSEPp
A
CitWAXp
B
CitUNKp
C
CitJuSacp
D
CitVO1/2p
E
PamMybAp
F
0
ATG
0
ATG
0
ATG
0
ATG
0
ATG
0
ATG
Additional file 1: Fig. S1
Additional file 1; Fig S1. Schematic representation of the promoter region.
The various cis elements identified in the candidate promoters CitSEPp, CitWAXp, CitUNKp, CitJuSacp, CitVO1p and Plum PamMybAp are shown as colored bars, respectively. The entire nucleotide sequences are available from the GenBank under the accession numbers CitSEPp (MK012379), CitWAXp (MK012380), CitUNKp (MK012381), CitJuSacp (MK012382), CitVO1p (MK012383), CitVO2p (MK012384), PamMybAp (MK012380).

## Slide 2
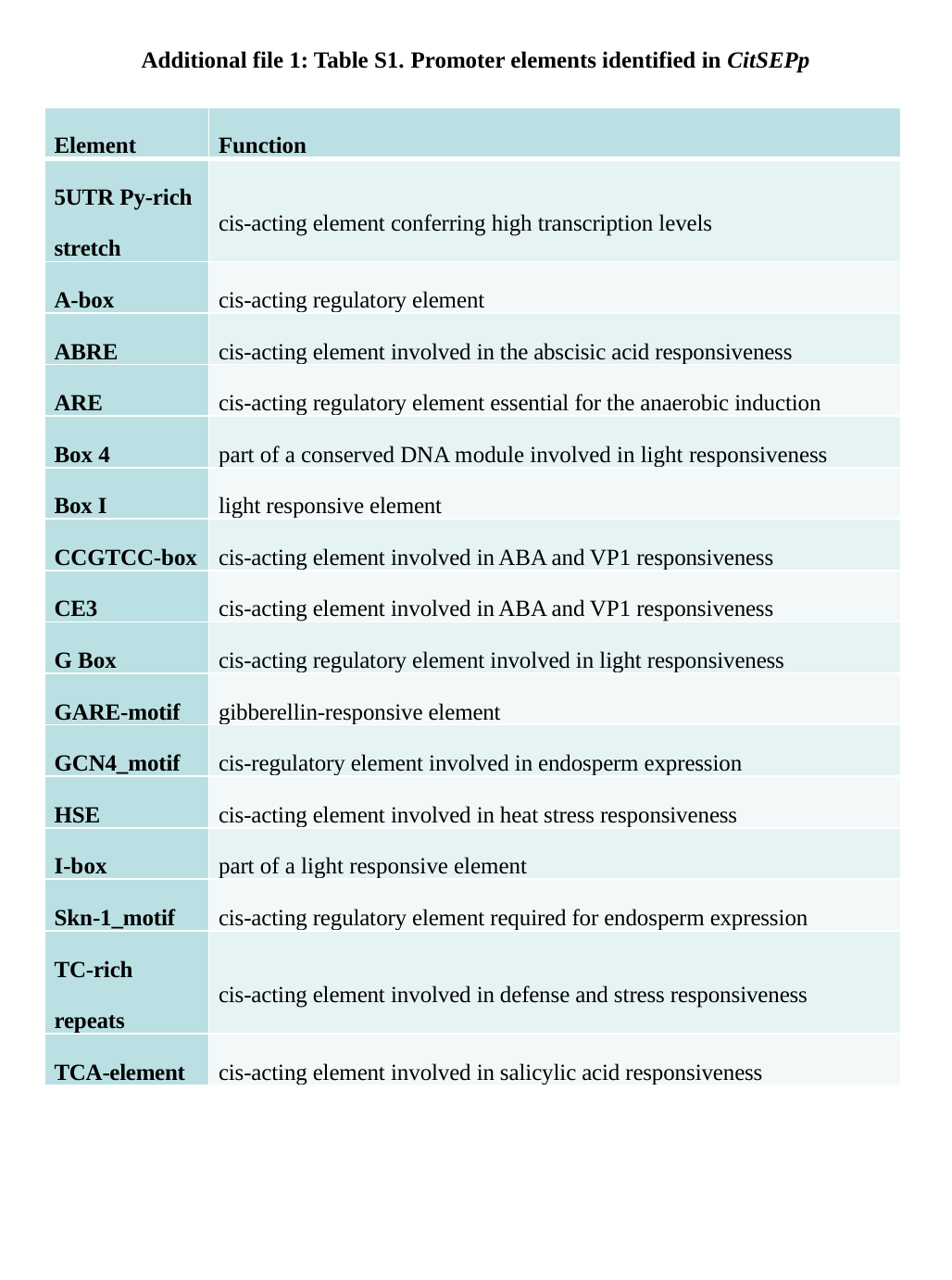

Additional file 1: Table S1. Promoter elements identified in CitSEPp
| Element | Function |
| --- | --- |
| 5UTR Py-rich stretch | cis-acting element conferring high transcription levels |
| A-box | cis-acting regulatory element |
| ABRE | cis-acting element involved in the abscisic acid responsiveness |
| ARE | cis-acting regulatory element essential for the anaerobic induction |
| Box 4 | part of a conserved DNA module involved in light responsiveness |
| Box I | light responsive element |
| CCGTCC-box | cis-acting element involved in ABA and VP1 responsiveness |
| CE3 | cis-acting element involved in ABA and VP1 responsiveness |
| G Box | cis-acting regulatory element involved in light responsiveness |
| GARE-motif | gibberellin-responsive element |
| GCN4\_motif | cis-regulatory element involved in endosperm expression |
| HSE | cis-acting element involved in heat stress responsiveness |
| I-box | part of a light responsive element |
| Skn-1\_motif | cis-acting regulatory element required for endosperm expression |
| TC-rich repeats | cis-acting element involved in defense and stress responsiveness |
| TCA-element | cis-acting element involved in salicylic acid responsiveness |

## Slide 3
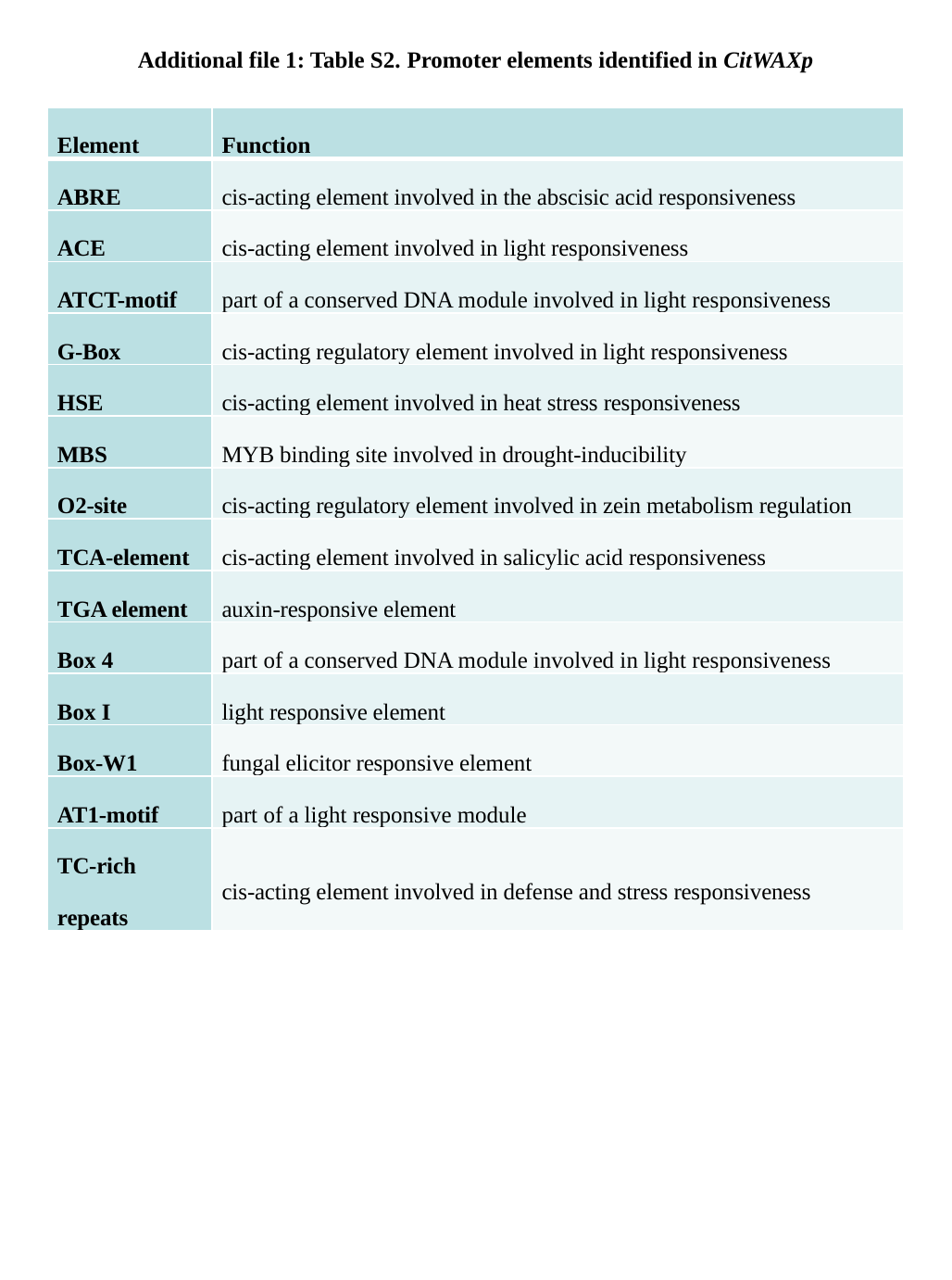

Additional file 1: Table S2. Promoter elements identified in CitWAXp
| Element | Function |
| --- | --- |
| ABRE | cis-acting element involved in the abscisic acid responsiveness |
| ACE | cis-acting element involved in light responsiveness |
| ATCT-motif | part of a conserved DNA module involved in light responsiveness |
| G-Box | cis-acting regulatory element involved in light responsiveness |
| HSE | cis-acting element involved in heat stress responsiveness |
| MBS | MYB binding site involved in drought-inducibility |
| O2-site | cis-acting regulatory element involved in zein metabolism regulation |
| TCA-element | cis-acting element involved in salicylic acid responsiveness |
| TGA element | auxin-responsive element |
| Box 4 | part of a conserved DNA module involved in light responsiveness |
| Box I | light responsive element |
| Box-W1 | fungal elicitor responsive element |
| AT1-motif | part of a light responsive module |
| TC-rich repeats | cis-acting element involved in defense and stress responsiveness |

## Slide 4
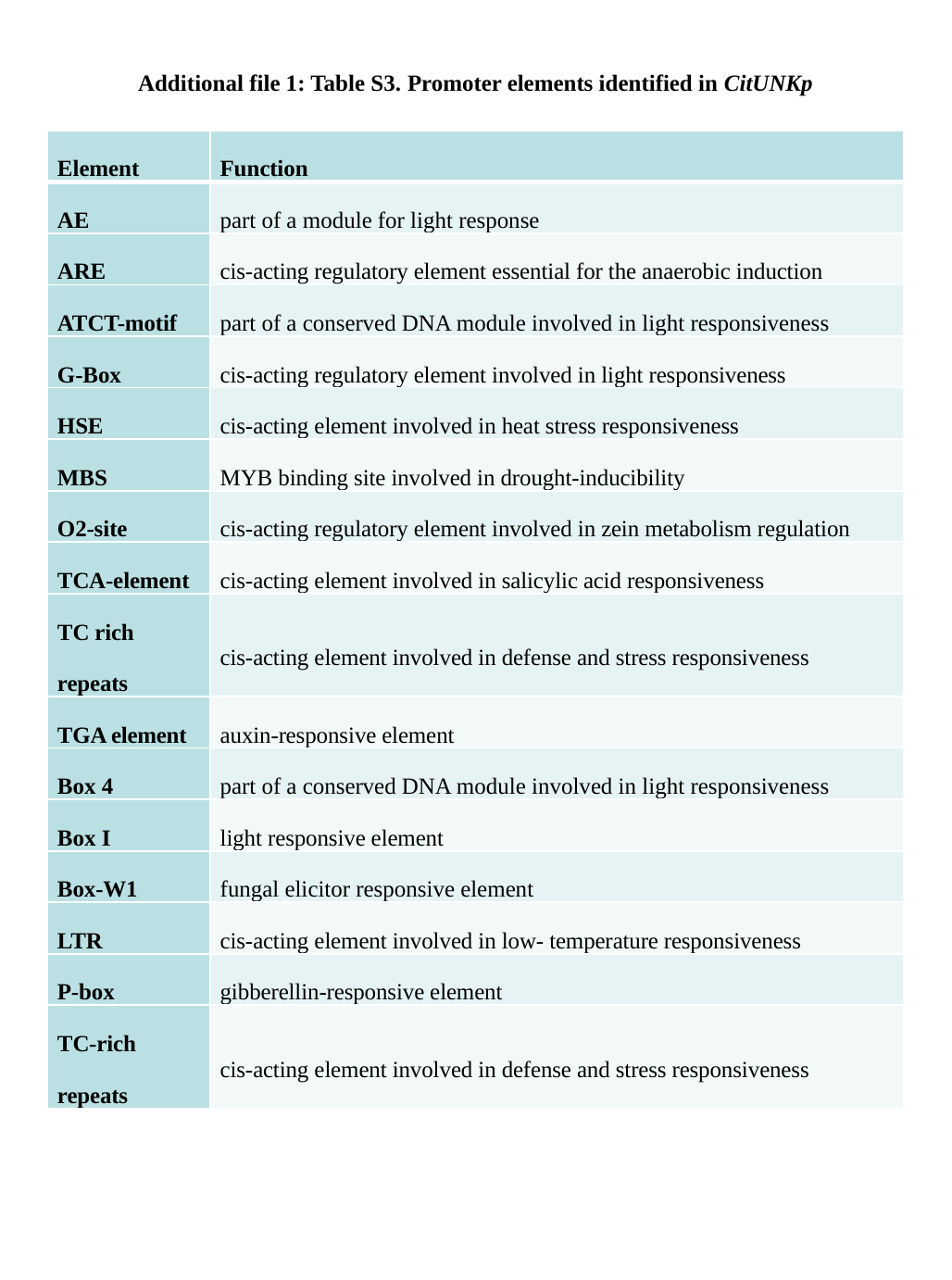

Additional file 1: Table S3. Promoter elements identified in CitUNKp
| Element | Function |
| --- | --- |
| AE | part of a module for light response |
| ARE | cis-acting regulatory element essential for the anaerobic induction |
| ATCT-motif | part of a conserved DNA module involved in light responsiveness |
| G-Box | cis-acting regulatory element involved in light responsiveness |
| HSE | cis-acting element involved in heat stress responsiveness |
| MBS | MYB binding site involved in drought-inducibility |
| O2-site | cis-acting regulatory element involved in zein metabolism regulation |
| TCA-element | cis-acting element involved in salicylic acid responsiveness |
| TC rich repeats | cis-acting element involved in defense and stress responsiveness |
| TGA element | auxin-responsive element |
| Box 4 | part of a conserved DNA module involved in light responsiveness |
| Box I | light responsive element |
| Box-W1 | fungal elicitor responsive element |
| LTR | cis-acting element involved in low- temperature responsiveness |
| P-box | gibberellin-responsive element |
| TC-rich repeats | cis-acting element involved in defense and stress responsiveness |

## Slide 5
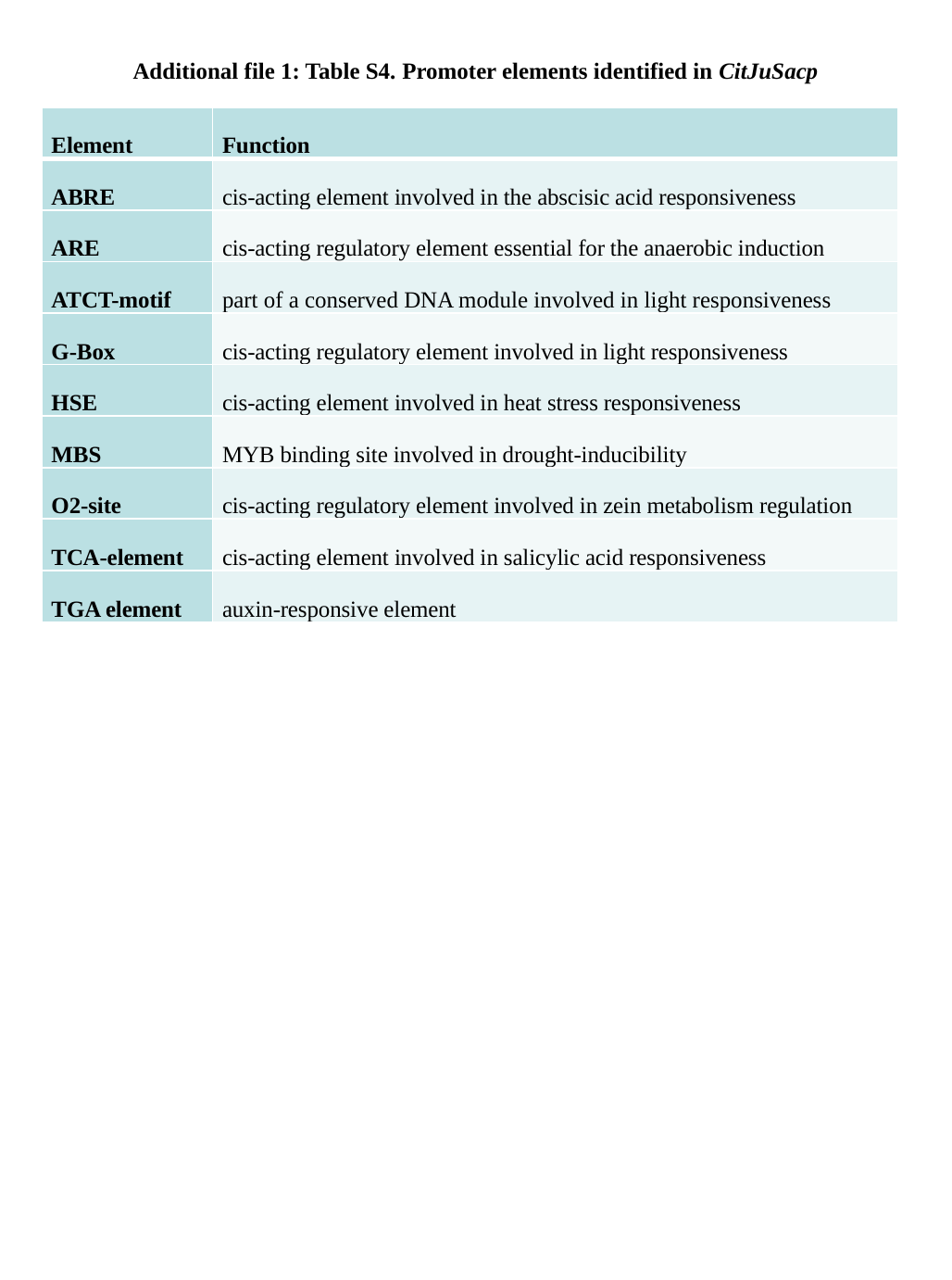

Additional file 1: Table S4. Promoter elements identified in CitJuSacp
| Element | Function |
| --- | --- |
| ABRE | cis-acting element involved in the abscisic acid responsiveness |
| ARE | cis-acting regulatory element essential for the anaerobic induction |
| ATCT-motif | part of a conserved DNA module involved in light responsiveness |
| G-Box | cis-acting regulatory element involved in light responsiveness |
| HSE | cis-acting element involved in heat stress responsiveness |
| MBS | MYB binding site involved in drought-inducibility |
| O2-site | cis-acting regulatory element involved in zein metabolism regulation |
| TCA-element | cis-acting element involved in salicylic acid responsiveness |
| TGA element | auxin-responsive element |

## Slide 6
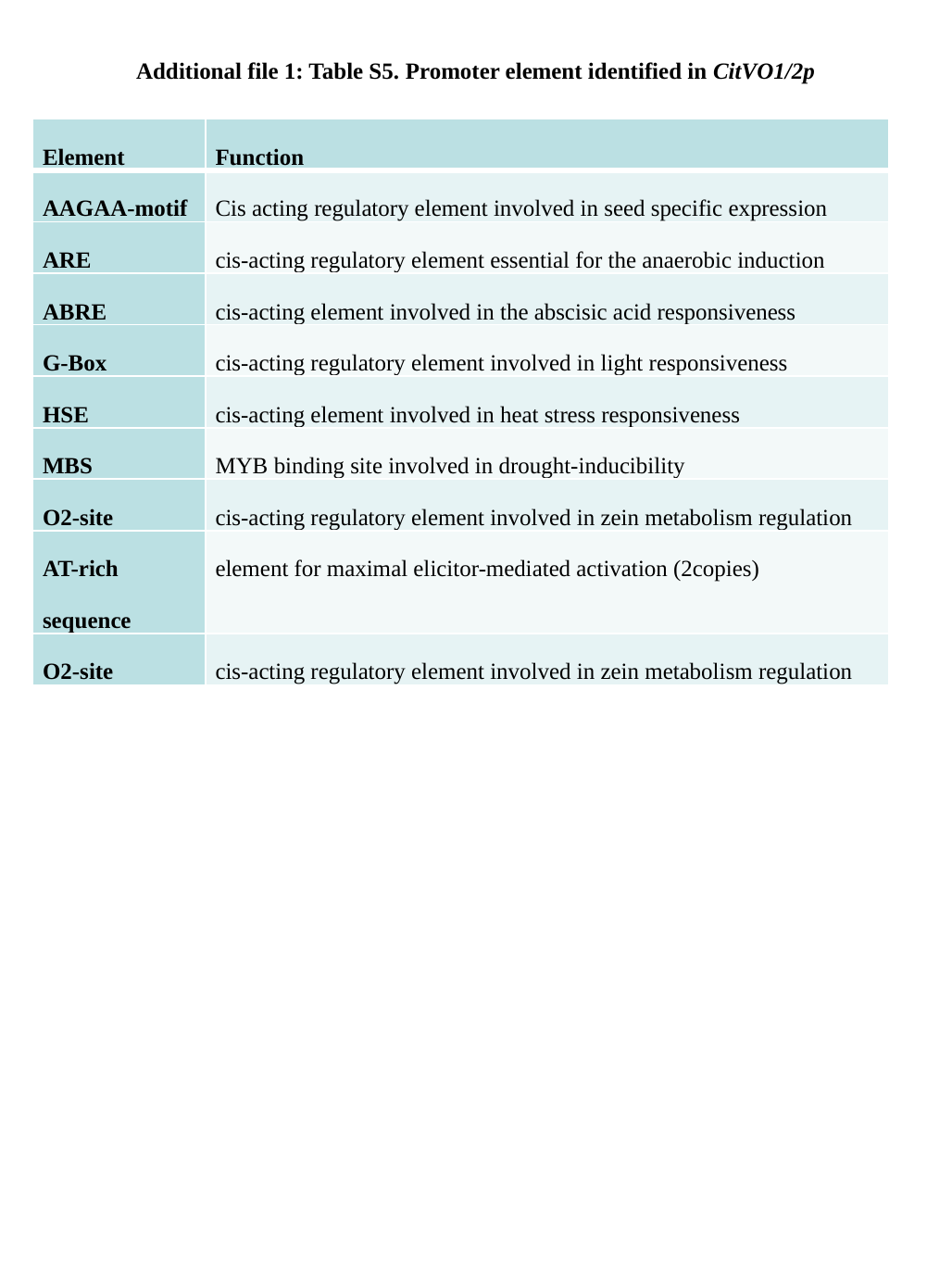

Additional file 1: Table S5. Promoter element identified in CitVO1/2p
| Element | Function |
| --- | --- |
| AAGAA-motif | Cis acting regulatory element involved in seed specific expression |
| ARE | cis-acting regulatory element essential for the anaerobic induction |
| ABRE | cis-acting element involved in the abscisic acid responsiveness |
| G-Box | cis-acting regulatory element involved in light responsiveness |
| HSE | cis-acting element involved in heat stress responsiveness |
| MBS | MYB binding site involved in drought-inducibility |
| O2-site | cis-acting regulatory element involved in zein metabolism regulation |
| AT-rich sequence | element for maximal elicitor-mediated activation (2copies) |
| O2-site | cis-acting regulatory element involved in zein metabolism regulation |

## Slide 7
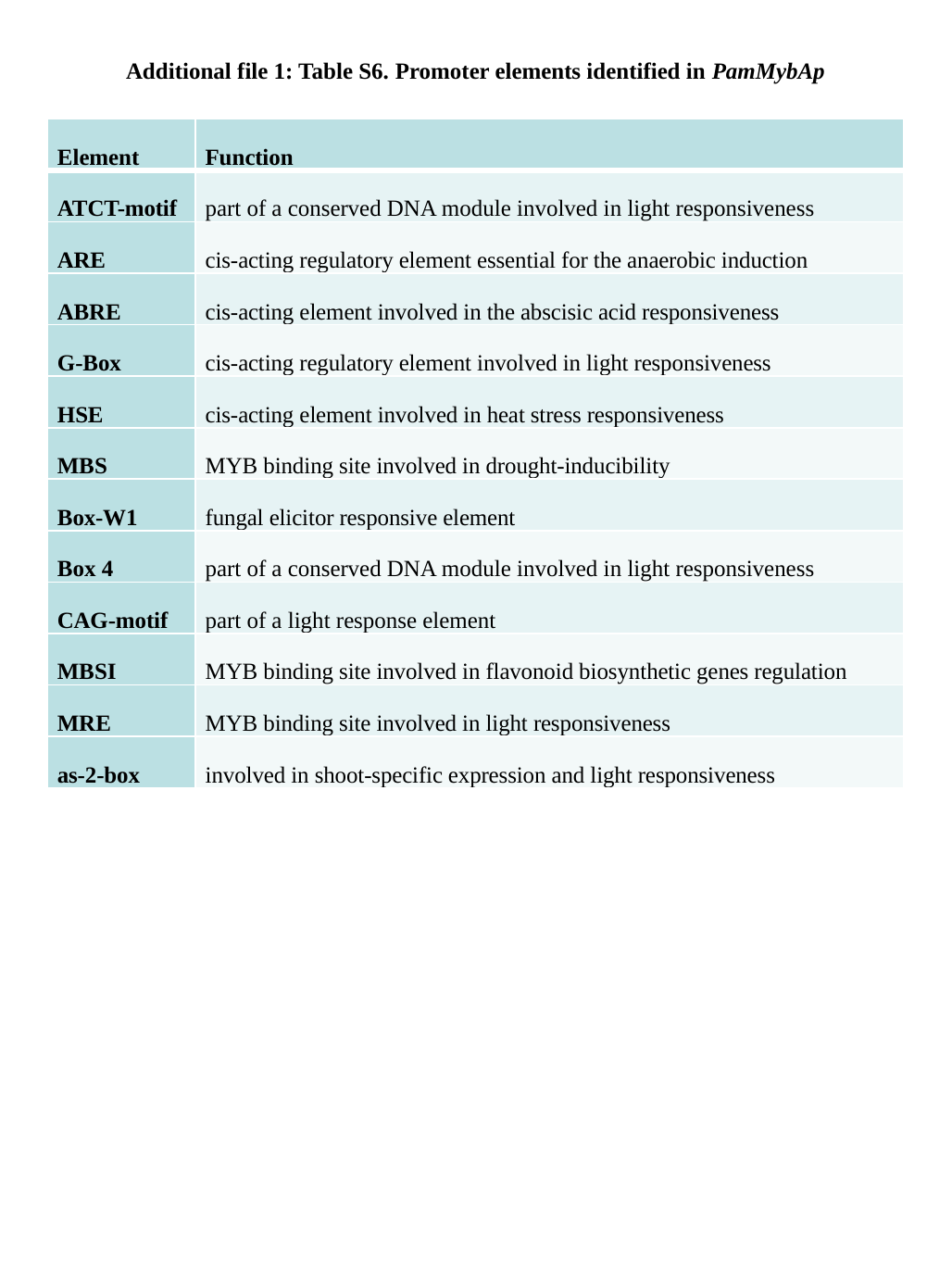

Additional file 1: Table S6. Promoter elements identified in PamMybAp
| Element | Function |
| --- | --- |
| ATCT-motif | part of a conserved DNA module involved in light responsiveness |
| ARE | cis-acting regulatory element essential for the anaerobic induction |
| ABRE | cis-acting element involved in the abscisic acid responsiveness |
| G-Box | cis-acting regulatory element involved in light responsiveness |
| HSE | cis-acting element involved in heat stress responsiveness |
| MBS | MYB binding site involved in drought-inducibility |
| Box-W1 | fungal elicitor responsive element |
| Box 4 | part of a conserved DNA module involved in light responsiveness |
| CAG-motif | part of a light response element |
| MBSI | MYB binding site involved in flavonoid biosynthetic genes regulation |
| MRE | MYB binding site involved in light responsiveness |
| as-2-box | involved in shoot-specific expression and light responsiveness |
