# Supplementary material for: Isolation of novel citrus and plum fruit promoters and their functional characterization for fruit biotechnology
Source: BMC Biotechnol. 2020 Aug 20;20:43. doi: 10.1186/s12896-020-00635-w (PMC7439555; doi:10.1186/s12896-020-00635-w)
Supplement: Supplementary file 2 — Additional file 2: Table S1. Transgene copy number measurements in T1 sibling transgenic Micro-Tom tomato lines from 3 independent events (4 plants from each family). Table S2. ddPCR primers and probe for reference and transgene detection. [file 12896_2020_635_MOESM2_ESM.pptx]

## Slide 1
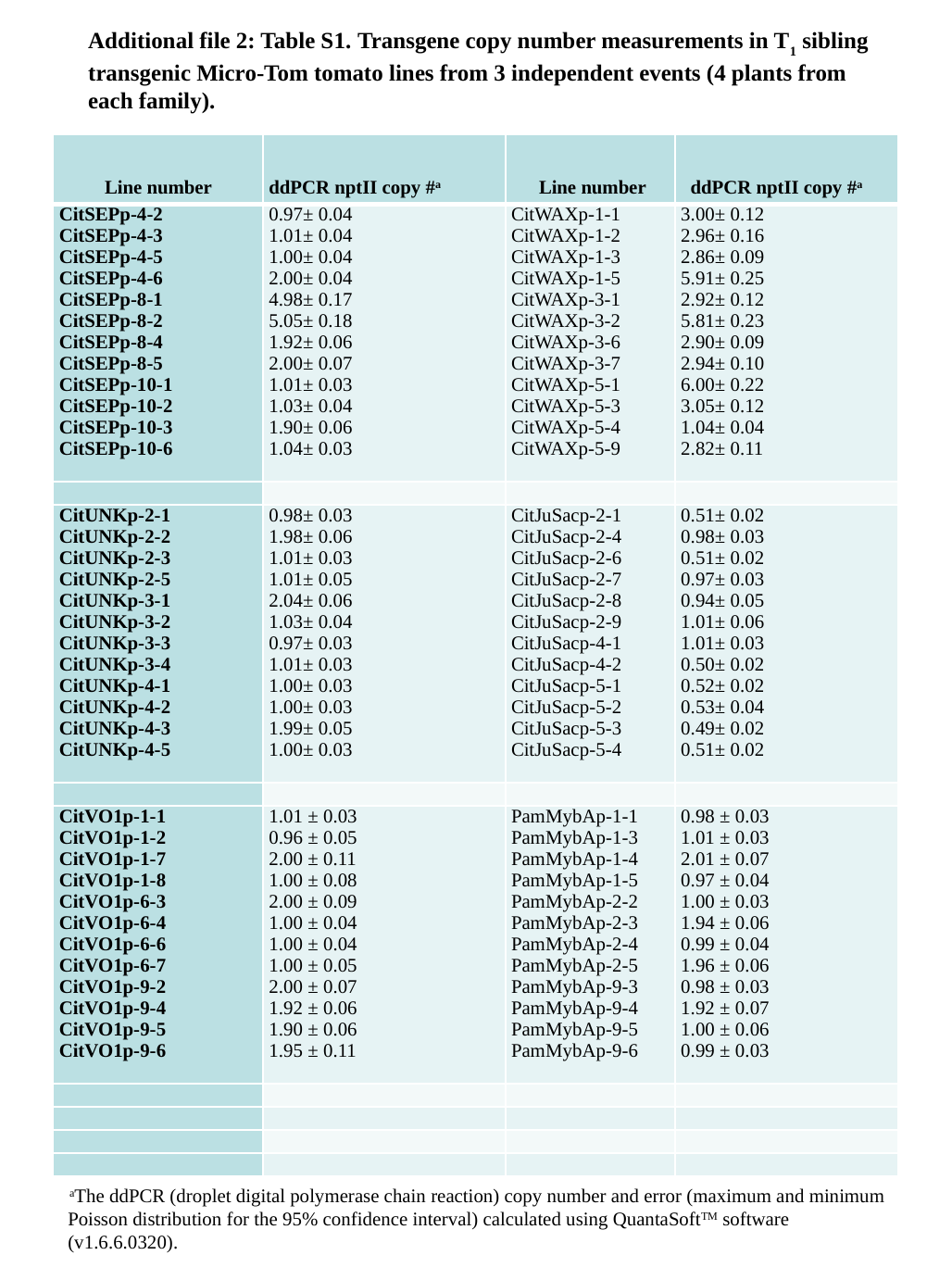

Additional file 2: Table S1. Transgene copy number measurements in T1 sibling transgenic Micro-Tom tomato lines from 3 independent events (4 plants from each family).
| Line number | ddPCR nptII copy #a | Line number | ddPCR nptII copy #a |
| --- | --- | --- | --- |
| CitSEPp-4-2 CitSEPp-4-3 CitSEPp-4-5 CitSEPp-4-6 CitSEPp-8-1 CitSEPp-8-2 CitSEPp-8-4 CitSEPp-8-5 CitSEPp-10-1 CitSEPp-10-2 CitSEPp-10-3 CitSEPp-10-6 | 0.97± 0.04 1.01± 0.04 1.00± 0.04 2.00± 0.04 4.98± 0.17 5.05± 0.18 1.92± 0.06 2.00± 0.07 1.01± 0.03 1.03± 0.04 1.90± 0.06 1.04± 0.03 | CitWAXp-1-1 CitWAXp-1-2 CitWAXp-1-3 CitWAXp-1-5 CitWAXp-3-1 CitWAXp-3-2 CitWAXp-3-6 CitWAXp-3-7 CitWAXp-5-1 CitWAXp-5-3 CitWAXp-5-4 CitWAXp-5-9 | 3.00± 0.12 2.96± 0.16 2.86± 0.09 5.91± 0.25 2.92± 0.12 5.81± 0.23 2.90± 0.09 2.94± 0.10 6.00± 0.22 3.05± 0.12 1.04± 0.04 2.82± 0.11 |
| | | | |
| CitUNKp-2-1 CitUNKp-2-2 CitUNKp-2-3 CitUNKp-2-5 CitUNKp-3-1 CitUNKp-3-2 CitUNKp-3-3 CitUNKp-3-4 CitUNKp-4-1 CitUNKp-4-2 CitUNKp-4-3 CitUNKp-4-5 | 0.98± 0.03 1.98± 0.06 1.01± 0.03 1.01± 0.05 2.04± 0.06 1.03± 0.04 0.97± 0.03 1.01± 0.03 1.00± 0.03 1.00± 0.03 1.99± 0.05 1.00± 0.03 | CitJuSacp-2-1 CitJuSacp-2-4 CitJuSacp-2-6 CitJuSacp-2-7 CitJuSacp-2-8 CitJuSacp-2-9 CitJuSacp-4-1 CitJuSacp-4-2 CitJuSacp-5-1 CitJuSacp-5-2 CitJuSacp-5-3 CitJuSacp-5-4 | 0.51± 0.02 0.98± 0.03 0.51± 0.02 0.97± 0.03 0.94± 0.05 1.01± 0.06 1.01± 0.03 0.50± 0.02 0.52± 0.02 0.53± 0.04 0.49± 0.02 0.51± 0.02 |
| | | | |
| CitVO1p-1-1 CitVO1p-1-2 CitVO1p-1-7 CitVO1p-1-8 CitVO1p-6-3 CitVO1p-6-4 CitVO1p-6-6 CitVO1p-6-7 CitVO1p-9-2 CitVO1p-9-4 CitVO1p-9-5 CitVO1p-9-6 | 1.01 ± 0.03 0.96 ± 0.05 2.00 ± 0.11 1.00 ± 0.08 2.00 ± 0.09 1.00 ± 0.04 1.00 ± 0.04 1.00 ± 0.05 2.00 ± 0.07 1.92 ± 0.06 1.90 ± 0.06 1.95 ± 0.11 | PamMybAp-1-1 PamMybAp-1-3 PamMybAp-1-4 PamMybAp-1-5 PamMybAp-2-2 PamMybAp-2-3 PamMybAp-2-4 PamMybAp-2-5 PamMybAp-9-3 PamMybAp-9-4 PamMybAp-9-5 PamMybAp-9-6 | 0.98 ± 0.03 1.01 ± 0.03 2.01 ± 0.07 0.97 ± 0.04 1.00 ± 0.03 1.94 ± 0.06 0.99 ± 0.04 1.96 ± 0.06 0.98 ± 0.03 1.92 ± 0.07 1.00 ± 0.06 0.99 ± 0.03 |
| | | | |
| | | | |
| | | | |
| | | | |
aThe ddPCR (droplet digital polymerase chain reaction) copy number and error (maximum and minimum Poisson distribution for the 95% confidence interval) calculated using QuantaSoftTM software (v1.6.6.0320).

## Slide 2
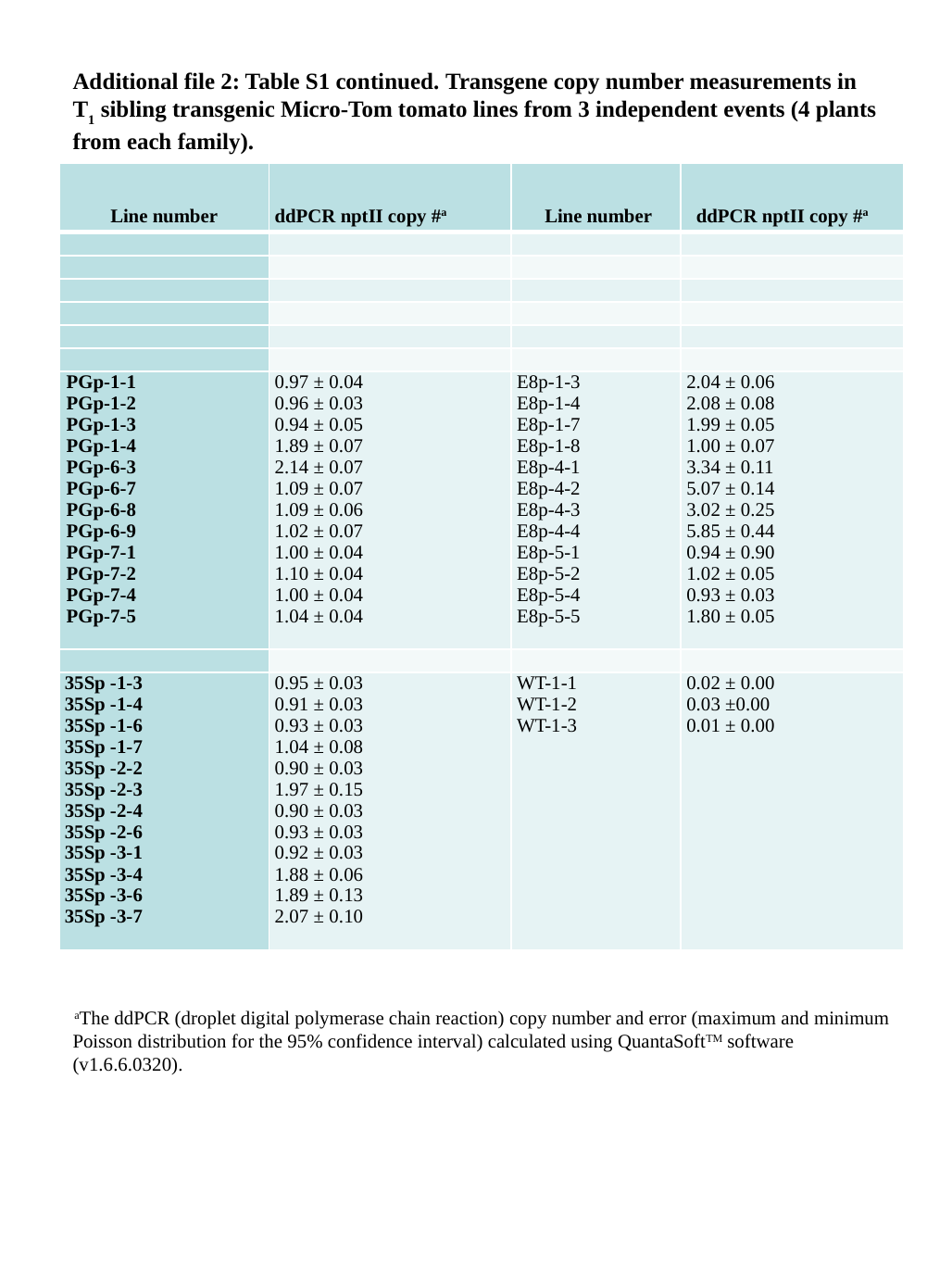

Additional file 2: Table S1 continued. Transgene copy number measurements in T1 sibling transgenic Micro-Tom tomato lines from 3 independent events (4 plants from each family).
| Line number | ddPCR nptII copy #a | Line number | ddPCR nptII copy #a |
| --- | --- | --- | --- |
| | | | |
| | | | |
| | | | |
| | | | |
| | | | |
| | | | |
| PGp-1-1 PGp-1-2 PGp-1-3 PGp-1-4 PGp-6-3 PGp-6-7 PGp-6-8 PGp-6-9 PGp-7-1 PGp-7-2 PGp-7-4 PGp-7-5 | 0.97 ± 0.04 0.96 ± 0.03 0.94 ± 0.05 1.89 ± 0.07 2.14 ± 0.07 1.09 ± 0.07 1.09 ± 0.06 1.02 ± 0.07 1.00 ± 0.04 1.10 ± 0.04 1.00 ± 0.04 1.04 ± 0.04 | E8p-1-3 E8p-1-4 E8p-1-7 E8p-1-8 E8p-4-1 E8p-4-2 E8p-4-3 E8p-4-4 E8p-5-1 E8p-5-2 E8p-5-4 E8p-5-5 | 2.04 ± 0.06 2.08 ± 0.08 1.99 ± 0.05 1.00 ± 0.07 3.34 ± 0.11 5.07 ± 0.14 3.02 ± 0.25 5.85 ± 0.44 0.94 ± 0.90 1.02 ± 0.05 0.93 ± 0.03 1.80 ± 0.05 |
| | | | |
| 35Sp -1-3 35Sp -1-4 35Sp -1-6 35Sp -1-7 35Sp -2-2 35Sp -2-3 35Sp -2-4 35Sp -2-6 35Sp -3-1 35Sp -3-4 35Sp -3-6 35Sp -3-7 | 0.95 ± 0.03 0.91 ± 0.03 0.93 ± 0.03 1.04 ± 0.08 0.90 ± 0.03 1.97 ± 0.15 0.90 ± 0.03 0.93 ± 0.03 0.92 ± 0.03 1.88 ± 0.06 1.89 ± 0.13 2.07 ± 0.10 | WT-1-1 WT-1-2 WT-1-3 | 0.02 ± 0.00 0.03 ±0.00 0.01 ± 0.00 |
aThe ddPCR (droplet digital polymerase chain reaction) copy number and error (maximum and minimum Poisson distribution for the 95% confidence interval) calculated using QuantaSoftTM software (v1.6.6.0320).

## Slide 3
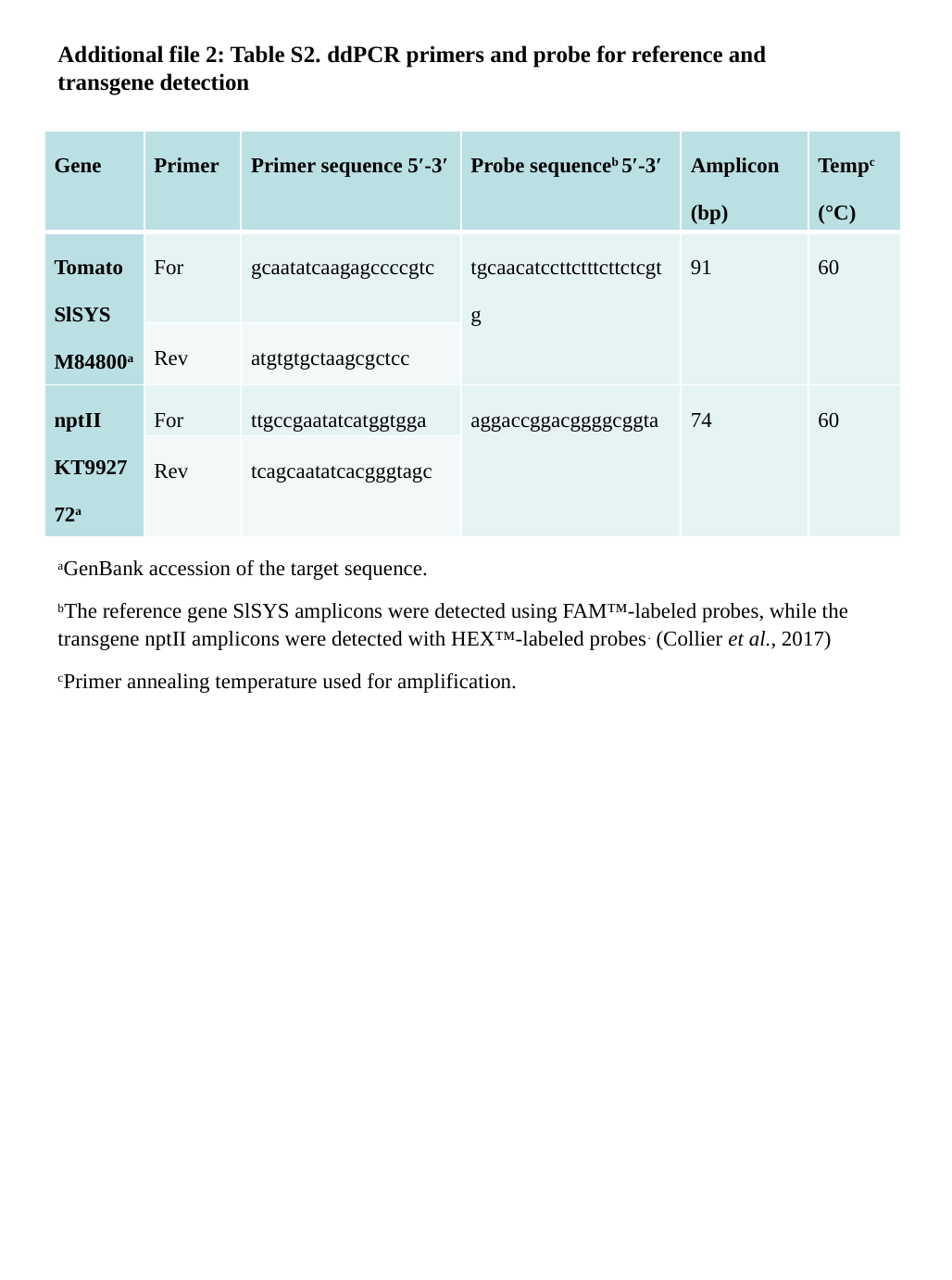

Additional file 2: Table S2. ddPCR primers and probe for reference and
transgene detection
| Gene | Primer | Primer sequence 5′-3′ | Probe sequenceb 5′-3′ | Amplicon (bp) | Tempc (°C) |
| --- | --- | --- | --- | --- | --- |
| Tomato SlSYS M84800a | For | gcaatatcaagagccccgtc | tgcaacatccttctttcttctcgtg | 91 | 60 |
| | Rev | atgtgtgctaagcgctcc | | | |
| nptII KT992772a | For | ttgccgaatatcatggtgga | aggaccggacggggcggta | 74 | 60 |
| | Rev | tcagcaatatcacgggtagc | | | |
aGenBank accession of the target sequence.
bThe reference gene SlSYS amplicons were detected using FAM™-labeled probes, while the transgene nptII amplicons were detected with HEX™-labeled probes. (Collier et al., 2017)
cPrimer annealing temperature used for amplification.
