# Supplementary material for: Isolation of novel citrus and plum fruit promoters and their functional characterization for fruit biotechnology
Source: BMC Biotechnol. 2020 Aug 20;20:43. doi: 10.1186/s12896-020-00635-w (PMC7439555; doi:10.1186/s12896-020-00635-w)
Supplement: Supplementary file 3 — Additional file 3: Figure S1. Histochemical staining of CitSEPp controlled GUSPlus activity in transgenic Arabidopsis tissues. (A) A whole seedling, (B) flowering inflorescence and (C) a developing silique stained for β-glucuronidase activity are shown. Figure S2. Histochemical staining of CitSEPp controlled GUSPlus activity in transgenic tobacco. Representative images for CitSEPp T0 transgenic tobacco (Nicotiana tabacum L. cv. Petit Havana SR1) tissues stained for β-glucuronidase activity are shown. (A) young leaf, (B) mature leaf (C) flowers with detectable staining in stigma and the flower base (ovule), circled. (D) stigma (E) bisected ovule [file 12896_2020_635_MOESM3_ESM.pptx]

## Slide 1
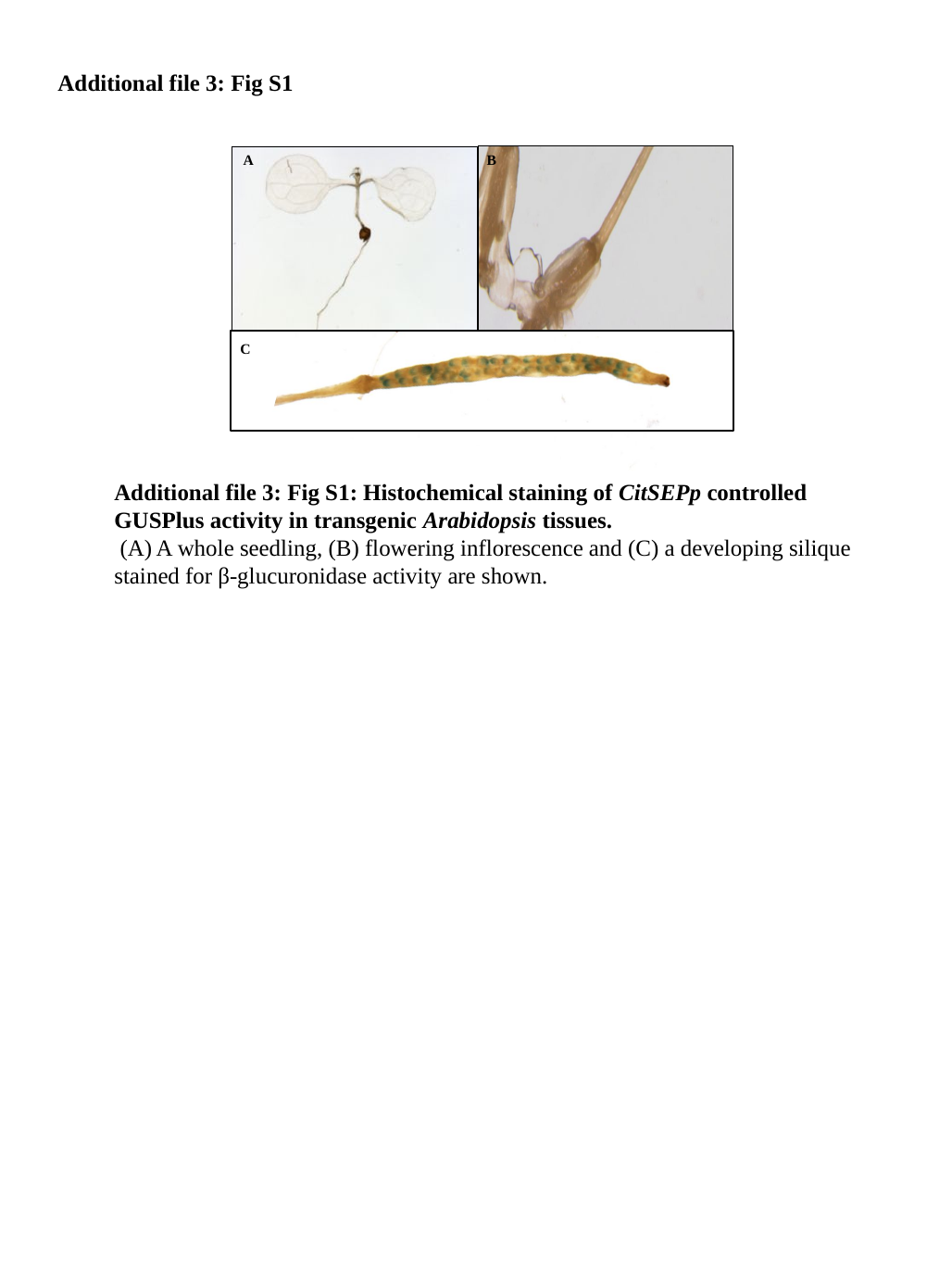

Additional file 3: Fig S1
A
B
C
Additional file 3: Fig S1: Histochemical staining of CitSEPp controlled GUSPlus activity in transgenic Arabidopsis tissues.
 (A) A whole seedling, (B) flowering inflorescence and (C) a developing silique stained for β-glucuronidase activity are shown.

## Slide 2
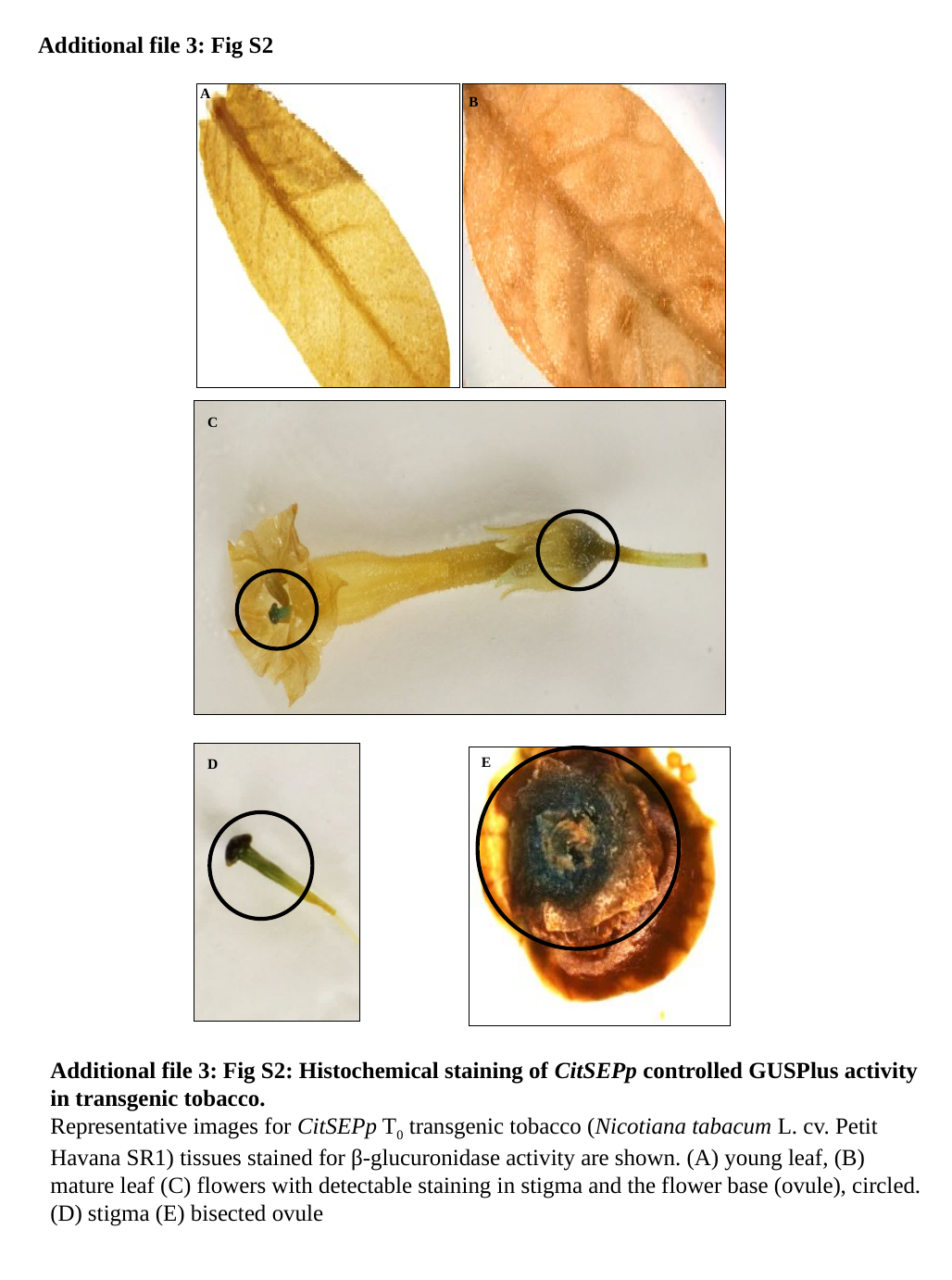

Additional file 3: Fig S2
A
B
C
E
D
Additional file 3: Fig S2: Histochemical staining of CitSEPp controlled GUSPlus activity in transgenic tobacco.
Representative images for CitSEPp T0 transgenic tobacco (Nicotiana tabacum L. cv. Petit Havana SR1) tissues stained for β-glucuronidase activity are shown. (A) young leaf, (B) mature leaf (C) flowers with detectable staining in stigma and the flower base (ovule), circled. (D) stigma (E) bisected ovule
